# *Pseudomonas aeruginosa* DEV phage exploits the essential LptD outer membrane protein as receptor for adsorption

**DOI:** 10.1128/mbio.03561-25

**Published:** 2026-01-22

**Authors:** Jimena Nieto Noblecia, Nathan F. Bellis, Cristian A. Antichi, Shirin Aminian, Francesca Forti, Federica A. Falchi, Davide Sposato, Francesco Imperi, Gino Cingolani, Federica Briani

**Affiliations:** 1 Dipartimento di Bioscienze, Università degli Studi di Milano 98853 , Milan, Italy; 2 Department of Biochemistry and Molecular Genetics, University of Alabama at Birmingham 164493, Birmingham, Alabama, USA; 3 Dipartimento di Scienze, Università degli Studi Roma Tre 164756 , Rome, Italy; Georgia Institute of Technology, Atlanta, Georgia, USA

**Keywords:** bacteriophage genetics, bacteriophage evolution, bacteriophage therapy, bacteriophage receptor, bacteriophage structure

## Abstract

*Pseudomonas aeruginosa* bacteriophage (phage) DEV is a podovirus of the *Schitoviridae* family, related to the prototypical *Escherichia coli* phage N4. N4 uses the novel glycan receptor (NGR) surface glycan, presumably bound by the gp66 appendages, and the NGR transporter NfrA, recognized by the phage gp65 tail sheath, as receptors for adsorption. In contrast, DEV relies on the O-antigen moiety of lipopolysaccharide (LPS) as the primary receptor recognized by the gp53 long tail fibers. However, DEV can infect deep-rough strains that lack the O-antigen moiety by using another, still unknown receptor. Here, we provide evidence that the essential LPS transporter LptD serves as the DEV secondary receptor and that DEV gp54 is its cognate receptor-binding protein. gp54 is encoded within the essential *gp56-gp55-gp54* operon, which also includes *gp56*, the short tail fiber gene. Using cryogenic electron microscopy, AlphaFold modeling, and genetic analysis, we show that DEV gp56, gp55, and gp54 assemble into a receptor-binding fiber (RBF) positioned laterally to a previously uncharacterized tail plug protein, gp74. The DEV RBF is functionally equivalent to the N4 sheath protein gp65, which associates with the tail plug gp53. Thus, DEV and N4 both use a glycan and its surface-exposing transporter as receptors for adsorption. To our knowledge, this is the first example of a *P. aeruginosa* phage using an essential outer membrane protein as a receptor, with implications for phage therapy.

**IMPORTANCE:**

*Pseudomonas aeruginosa* phage DEV uses the O-antigen of lipopolysaccharide as its primary receptor. In this study, we found that LptD, an essential and highly conserved outer membrane protein, serves as the secondary receptor for DEV. This interaction is mediated by a specialized receptor-binding fiber composed of the DEV proteins *gp54*, *gp55*, and *gp56*. We posit that the *gp56-gp55-gp54* genes form a functional module, possibly disseminated via horizontal gene transfer among distantly related phages, involved in tail sealing and the regulated unplugging of the tail upon interaction with the bacterial receptor. Given the high conservation of receptor-binding proteins among phages in the DEV *Litunavirus* genus, we anticipate that other members of this genus may also use LptD as their receptor. Since *Litunaviruses* are actively explored for phage therapy, insights into the interaction between DEV and its receptors could help develop more effective and targeted phage-based treatments.

## INTRODUCTION

Bacteriophage (phage) infection begins with the recognition of a surface-exposed bacterial molecule, the primary receptor, by a phage receptor-binding protein (RBP). In Gram-negative bacteria, this receptor is often the lipopolysaccharide (LPS), a complex glycolipid located in the outer leaflet of the outer membrane (OM) (1–4). The LPS consists of three parts: the lipid A, the innermost and highly conserved portion; the core oligosaccharide, with low intraspecies variability; and the O-antigen, a polymer of repeated oligosaccharide units with high intraspecies variability in composition and repeat number (5). This configuration, where the core is capped with the O-antigen, is termed “smooth” LPS in juxtaposition with “rough” and “deep-rough” LPS species lacking either the O-antigen only or the O-antigen and the outermost portion of the core oligosaccharide, respectively. Both the O-antigen and core moieties can act as receptors for phages infecting bacterial strains with smooth or rough LPS, respectively. Additionally, some LPS-binding phages possess a secondary receptor, typically an OM protein (OMP) (2, 4). In smooth strains, the OMPs may be concealed by the LPS polysaccharide portion extending above the OM layer; thus, the infecting phage must first interact with the LPS to reach the secondary receptor. In contrast, in rough strains with short LPS, secondary receptors may be exposed and accessible. The ability to recognize a secondary receptor broadens the host spectrum of phages exploiting the O-antigen as the primary receptor to include rough strains (6).

The utilization of two bacterial receptors belonging to different chemical classes does not necessarily imply that the phage employs different structures to bind them. For example, the prototypical *Escherichia coli* myovirus (i.e., a phage with a long contractile tail) T4 binds both the LPS and the OmpC porin with different portions of the long tail fibers (7–9). On the contrary, the *E. coli* siphovirus (i.e., a phage with a long non-contractile tail) T5 exploits the long tail fibers to bind the O-antigen and the tailspike at the tail tip to interact with the ferrichrome receptor FhuA (10–15).

In this work, we studied the adsorption of phage DEV to its *Pseudomonas aeruginosa* host. DEV is a podovirus, that is, a phage with a short, non-contractile tail, belonging to the *Schitoviridae* family like the *E. coli* phage N4 (16–18). DEV can propagate both in the smooth PAO1 *P. aeruginosa* strain and in deep-rough *galU*, *algC*, or *wapH* PAO1 mutants, but not in a *wzy* mutant producing an LPS species in which the core is capped with a single O-antigen repeat (19). A DEV Δ53 deletion mutant lacking the *gp53* tail fiber gene does not grow in wild-type (wt) PAO1 or *wzy* mutant, whereas it is still able to infect the *galU*, *algC*, or *wapH* deep-rough mutants (20). Based on these data, we postulated that (i) when infecting the smooth strain PAO1, DEV attaches to the O-antigen with the gp53 tail fibers, (ii) DEV has another receptor besides the O-antigen that is recognized by a phage RBP other than gp53, and (iii) the second phage receptor is accessible in deep-rough mutants but not in PAO1 or in the *wzy* mutant (20).

In this paper, we present evidence supporting LPS transporter LptD as the DEV secondary receptor and DEV gp54 as the phage’s second RBP. The *gp54* gene is part of an essential operon that includes the *gp56* gene, which encodes a short tail fiber we previously localized at the DEV tail tip by structural analysis (20). We provide structural evidence that DEV gp54, gp55, and gp56 form a specialized receptor-binding fiber (RBF) complex. Surprisingly, this complex attaches laterally to a newly identified plug, gp74, which is conserved in N4-like phages. The DEV RBF complex interacts with both the LPS transporter LptD through gp54 and with the plug gp74 via gp56, explaining why deletion of gp56 abolishes infectivity and causes loss of DNA from DEV virions.

## RESULTS

### LPS depletion stimulates DEV Δ53 adsorption

We explored the hypothesis that DEV, like other podoviruses, may first attach to the O-antigen and then exploit the LPS core oligosaccharide as a secondary receptor (4, 21). In this case, a strain that does not produce any LPS form should be non-permissive for DEV adsorption. To test this hypothesis, we used a PAO1 derivative with the *lpxA* gene under the control of the *E. coli araBp* promoter (22). In this mutant, transcription of *lpxA* is inducible with arabinose. *lpxA* encodes an essential enzyme of the lipid A biosynthetic pathway, and its depletion blocks the biosynthesis of all forms of LPS (i.e., uncapped and capped with either the common polysaccharide antigen or the O-antigen) (23–25).

**Fig 1:**
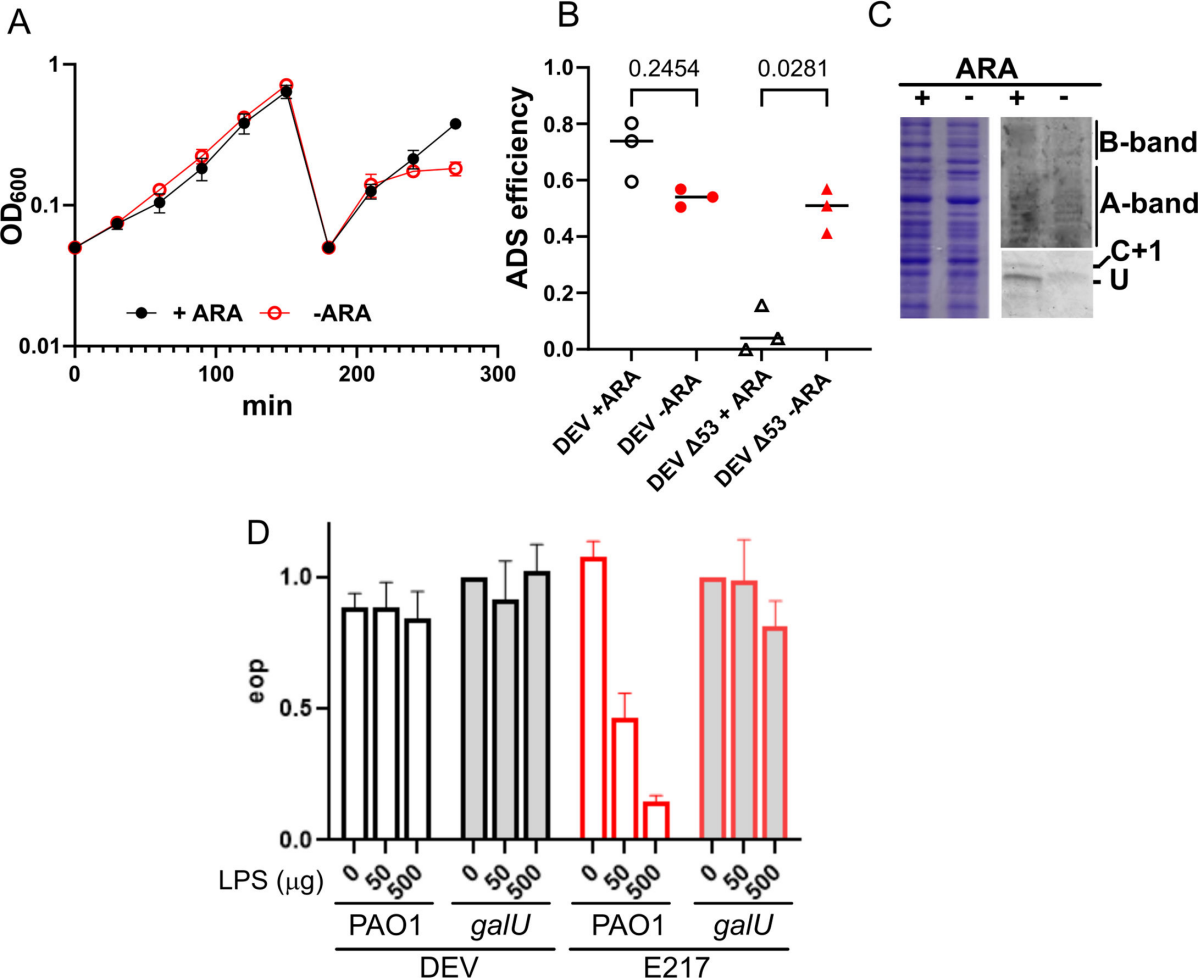
DEV adsorption upon LPS depletion. (**A**) Growth of the *araBp-lpxA* strain in presence or absence of 0.2% arabinose (ARA). The adsorption assay (**B**) and LPS analysis (**C**) were executed on bacteria collected at the last time point (270 min). (**B**) Adsorption (ADS) efficiency of DEV (circles) and DEV Δ53 (triangles) in the presence or absence of arabinose. The significance of the difference (P) estimated with one-way ANOVA and Šídák’s multiple comparisons test is reported. (**C**) LPS (right panels) and proteins (left panel) extracted from cultures grown in the presence or absence of arabinose. Proteins were analyzed by SDS-PAGE and Coomassie staining and LPS by 18% Tricine gel electrophoresis (lower panel) and 12% SDS-PAGE (upper panel) and emerald-green staining. LPS species were recognized based on their electrophoretic profile (19). B-band, serotype-specific LPS; A-band, common antigen LPS; C+1, LPS capped with a single O-antigen repeat; u, uncapped LPS. (**D**) Pre-incubation with LPS extracted from PAO1 (empty bars) inactivated E217 but not DEV. LPS extracted from the *galU* mutant (filled bars) did not impair infection by either phage. Bars indicate average efficiency of plating (EOP, *N* = 2) with range.

We found that in the absence of arabinose, *araBp-lpxA* PAO1 cultures stopped growing after five to six divisions, and the arrested cultures were highly depleted of LPS. However, DEV adsorption was only slightly affected (Fig. 1A through C). Interestingly, the gp53 tail fiber-defective mutant DEV Δ53, which does not adsorb to PAO1, could adsorb to LPS-depleted PAO1 with efficiency similar to that of the wt DEV (Fig. 1B), suggesting that the LPS is not required for DEV Δ53 infection.

We also tested whether DEV loses infectivity upon incubation with purified LPS, as it was observed for E217, a phage targeting the LPS O-antigen for adsorption (26, 27). Notably, incubation of DEV with the LPS extracted from PAO1 or from the *galU* mutant did not reduce phage infectivity (Fig. 1D).

Thus, the DEV secondary receptor is not an LPS species.

### Mutations in *lptD* and *rpoH* confer DEV resistance to the PAO1 *galU* mutant

**Fig 2:**
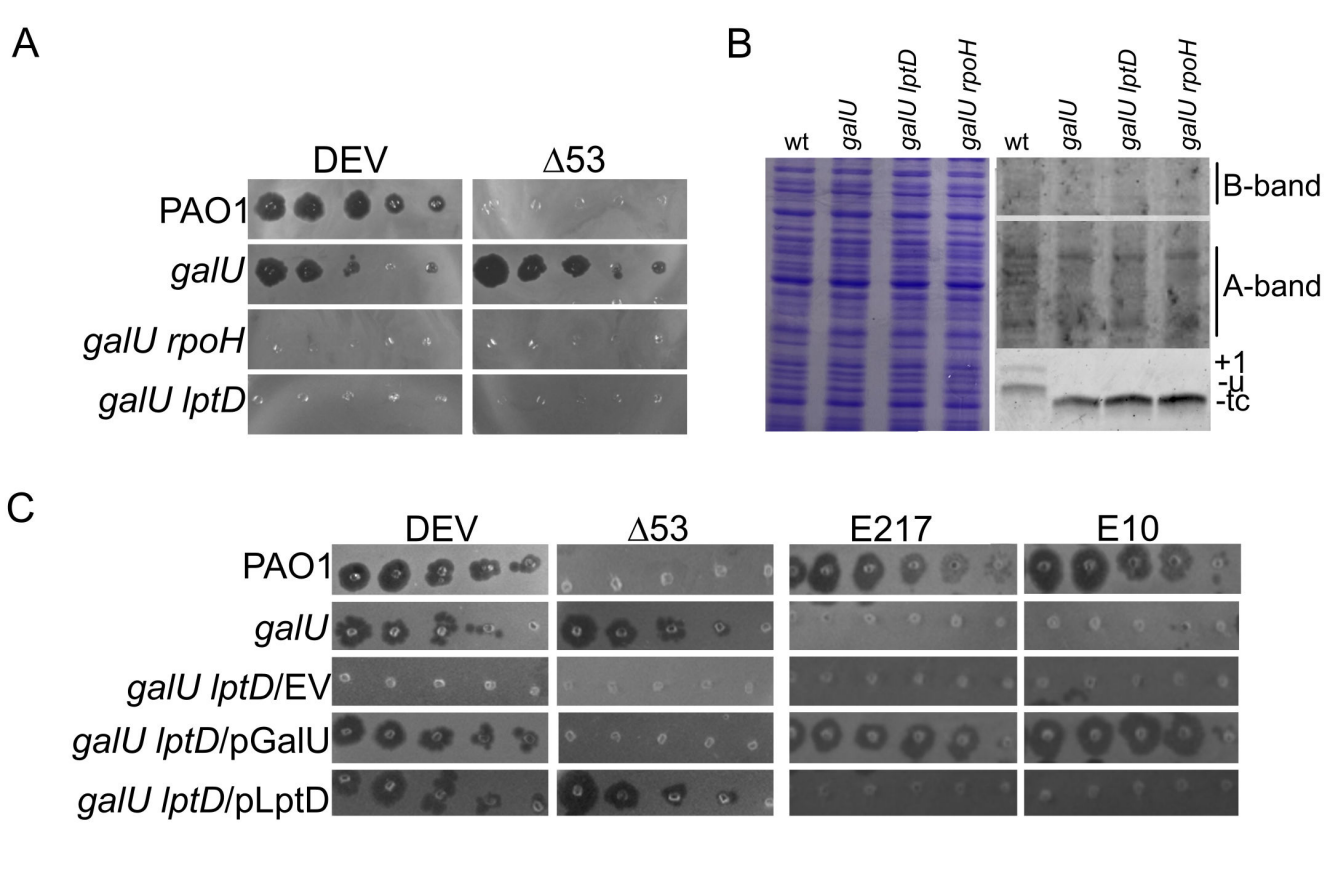
Effect of *lptD* and *rpoH* mutations on the LPS profile and phage growth. (**A and C**) Tenfold serial dilutions (from left to right) of the indicated phages were replicated on PAO1 or the PAO1 mutants indicated on the left of the panels. The plates were incubated for 16 h at 37°C. EV, empty vector. (**B**) LPS extracted from cultures of PAO1 (wt) or the PAO1 mutants indicated on top of the lanes, grown at 37°C. Samples corresponding to the same total OD_600_ were split and used to extract proteins as a sampling control (left panel) and LPS (right panels). Proteins and LPS were analyzed as described in the legend of Fig. 1. B-band, serotype-specific LPS; A-band, common antigen LPS; +1, LPS capped with a single O-antigen repeat; u, uncapped LPS; tc, LPS with truncated core oligosaccharide. The *galU lptD*^Δ33^ mutant is indicated as *galU lptD* in all panels.

To identify the secondary receptor of DEV, we searched for mutations that confer resistance to DEV in a *galU* defective strain, which lacks the DEV primary receptor, the O-antigen-capped LPS (19, 25). PAO1 *galU* cultures were plated on plates spread with DEV and the colonies tested for resistance to both wt DEV and *gp53*-defective DEV Δ53. The genomes of two independent (i.e., selected from independent bacterial cultures) *galU* derivatives resistant to both phages were sequenced to identify the mutation(s) responsible for phage resistance. Both mutants retained the *galU* frameshift mutation present in the parental strain (19) and had an additional mutation in either the *lptD* or *rpoH* gene, encoding the LPS transporter LptD or the alternative sigma factor σ^H^, respectively. Specifically, we found a 33 bp in-frame deletion (mutation *lptD^Δ33^*) that eliminates residues 880–890 in the 924 amino acids of LptD and a T>G substitution in *rpoH*, resulting in the replacement of the conserved valine at position 164 with glycine (V164G) in σ^H^. No other mutations were detected, implying that the mutations identified in *lptD* or *rpoH* are responsible for DEV resistance in deep-rough *galU* strains (Table 1; Fig. 2A and B).

**TABLE 1:** Efficiency of plating of DEV and DEV Δ53 on PAO1 double mutants

Mutant	DEV		DEV Δ53	
	Average^*a*^	SD^*b*^	Average^*a*^	SD^*b*^
PAO1	1.0	0.03	<0.003	n.a.
*galU*	0.9	0.07	1.0	0.11
*galU lptD* ^Δ33^	<0.0003	n.a.	<0.003	n.a.
*galU rpoH*	0.006	0.0005	<0.003	n.a.

^*a*^ Efficiency of plating (EOP) average (*N* = 3), determined as the ratio between the titer on each replicate culture and the average titer calculated on the reference strains (wt PAO1 for DEV, and PAO1 *galU* for DEV Δ53).

^*b*^ n.a., not applicable.

Since LptD is located in the OM, whereas σ^H^ is a cytoplasmic protein, we focused on LptD as a potential secondary receptor for DEV. To confirm that the mutation in *lptD* was responsible for the DEV-resistant phenotype of the *galU lptD*^Δ33^ mutant, we checked DEV plating efficiency on *galU lptD*^Δ33^ carrying the pLptD plasmid, which expresses the wt LptD protein. Both wt DEV and DEV Δ53 were able to grow in the complemented *galU lptD*^Δ33^/pLptD strain (Fig. 2C), confirming that the *lptD*^Δ33^ mutation is responsible for DEV resistance. We then tested DEV growth in the *galU lptD*^Δ33^ strain expressing wt GalU from the pGalU plasmid to assess whether the *lptD*^Δ33^ mutation caused DEV resistance only in the absence of GalU or also in its presence. DEV was able to form plaques on the complemented host, demonstrating that the *lptD^Δ33^* mutation does not block phage growth as long as the smooth LPS can be produced. Notably, E217 and E10 phages, which depend on the O-antigen for adsorption and do not infect the *galU* mutant (19, 26), were able to grow in the *galU lptD*^Δ33^/pGalU strain (Fig. 2C), suggesting that the *lptD*^Δ33^ mutation does not strongly impact LPS transport. Accordingly, no significant difference in the LPS level was observed between *galU* and *galU lptD*^Δ33^ mutants (Fig. 2B).

### DEV adsorption efficiency is low in the absence of the O-antigen and responds to LptD levels

**Fig 3:**
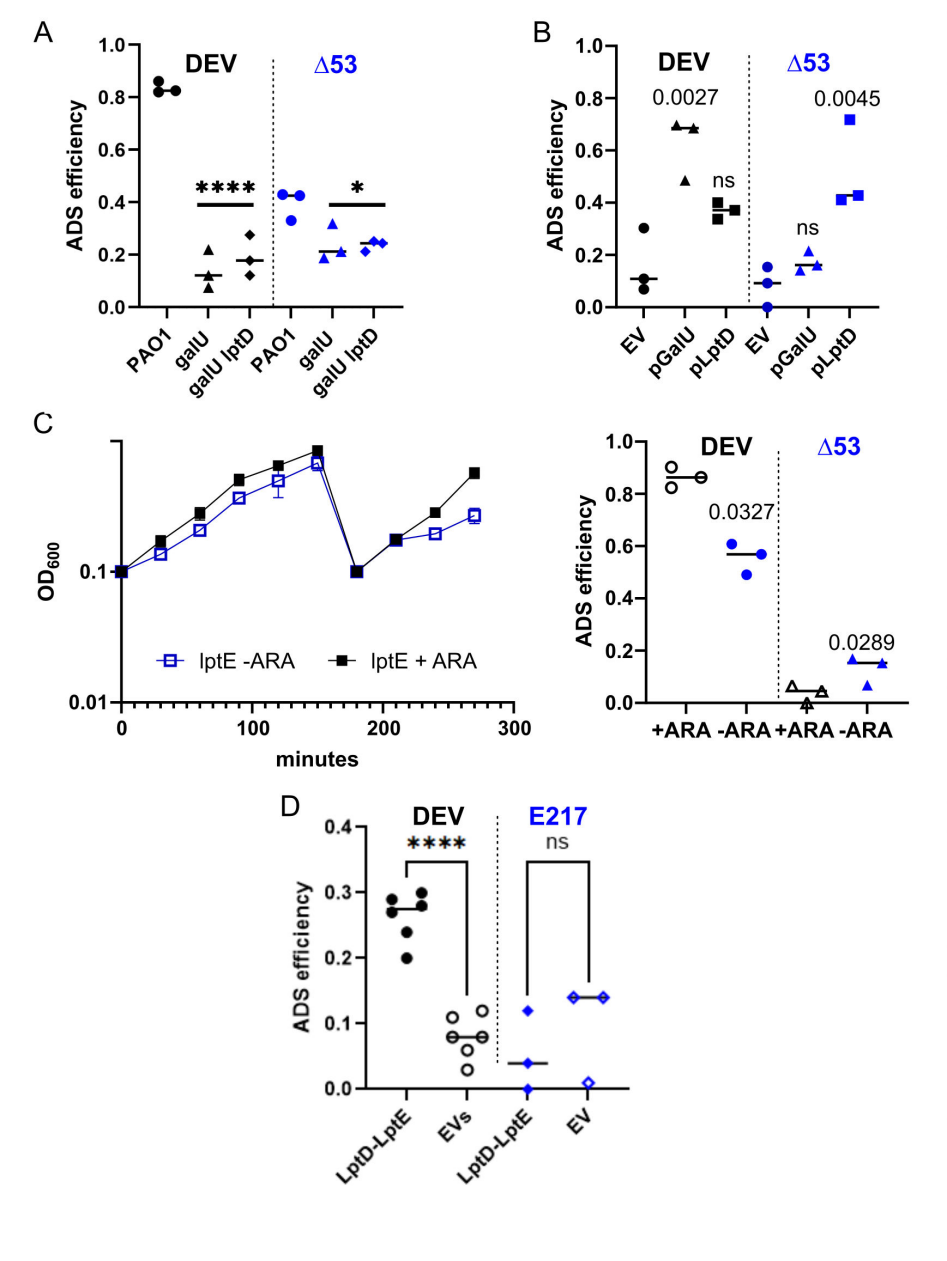
Efficiency of LptD-mediated DEV adsorption. Efficiency of adsorption to the indicated *P. aeruginosa* strains (**A**) or to the *galU lptD*^Δ33^ double mutant carrying the indicated plasmids (**B**) In panel A, the *galU lptD*^Δ33^ mutant is indicated as *galU lptD*. EV, empty vector, pGM931. The symbols represent replicate samples, and the lines represent the average. *P* was calculated with one-way ANOVA and Šídák’s multiple comparisons test. Only *P* values relative to the comparisons between PAO1 and the mutant strains (A, ****, <0.0001; *, <0.05) or between the control *galU lptD*^Δ33^/EV and the strains carrying the plasmids pGalU or pLptD (**B**) are reported for clarity. ns, not significant. (**C**) Growth of the *araBp-lptE* strain (left panel) and adsorption efficiency of DEV and DEV Δ53 (right panel) in the presence or absence of 0.2% arabinose (ARA). The adsorption assay was performed on bacteria collected at the last time point. The significance of the difference (*P*) estimated with a two-tailed *t*-test is reported. (**D**) Adsorption efficiency of DEV (circles) and E217 (diamonds) to *E. coli* Δ*waaG* expressing *P. aeruginosa* LptD and LptE from pLptD and pGM-lptE compatible plasmids (LptD-LptE). EVs, empty vectors. *P* was calculated with one-way ANOVA and Tukey *post hoc* test. ****, <0.0001; ns, not significant.

We measured the adsorption efficiency of DEV and DEV Δ53 to the *galU* and *galU lptD*^Δ33^ mutants compared with PAO1. Despite DEV’s high efficiency of plating (EOP) on the *galU* mutant, its adsorption to this strain was significantly reduced (from over 80% for PAO1 to 10%–15% for the *galU* mutant), similar to the adsorption to the DEV-resistant *galU lptD*^Δ33^ mutant. Accordingly, DEV Δ53 showed low adsorption to all tested strains (Fig. 3A). Complementation of the *galU* mutation with pGalU increased DEV adsorption to the *galU lptD*^Δ33^ mutant, but it did not improve Δ53 adsorption, as expected. In contrast, complementation of the *lptD*^Δ33^ mutation with pLptD similarly increased DEV and Δ53 adsorption to around 40%, with some fluctuations in the adsorption efficiency (Fig. 3B). Therefore, without the O-antigen or the O-antigen binding protein gp53, DEV adsorption becomes inefficient and/or unstable and depends on LptD levels.

We validated this finding by testing whether low LptD levels decreased DEV adsorption. Since LptE depletion prevents LptD insertion into the OM, we used a PAO1 derivative with arabinose-dependent *lptE* expression (strain *araBp-lptE*) (28). The mutant did not stop growing in the absence of arabinose, suggesting that LptE depletion was only partially achieved (Fig. 3C). In spite of this, DEV adsorption was reduced in *araBp-lptE* bacteria grown without arabinose, whereas, as expected, Δ53 adsorption to this smooth strain remained low in the presence or absence of arabinose (Fig. 3C). We then assessed whether DEV absorbed to an *E. coli* strain exposing *P. aeruginosa* LptD on the cell surface. We used a Δ*waaG::kan* deep-rough *E. coli* strain (29) expressing LptD and LptE of *P. aeruginosa* from compatible plasmids. We observed that DEV adsorption increased slightly but consistently in the presence of plasmids expressing *P. aeruginosa* proteins compared to the empty vectors. In contrast, E217 adsorption, which requires *P. aeruginosa* LPS (19, 26), remained similarly low in both strains (Fig. 3D). Overall, these results support the hypothesis that LptD is the DEV secondary receptor.

### The *gp56-55-54* operon is essential

**Fig 4:**
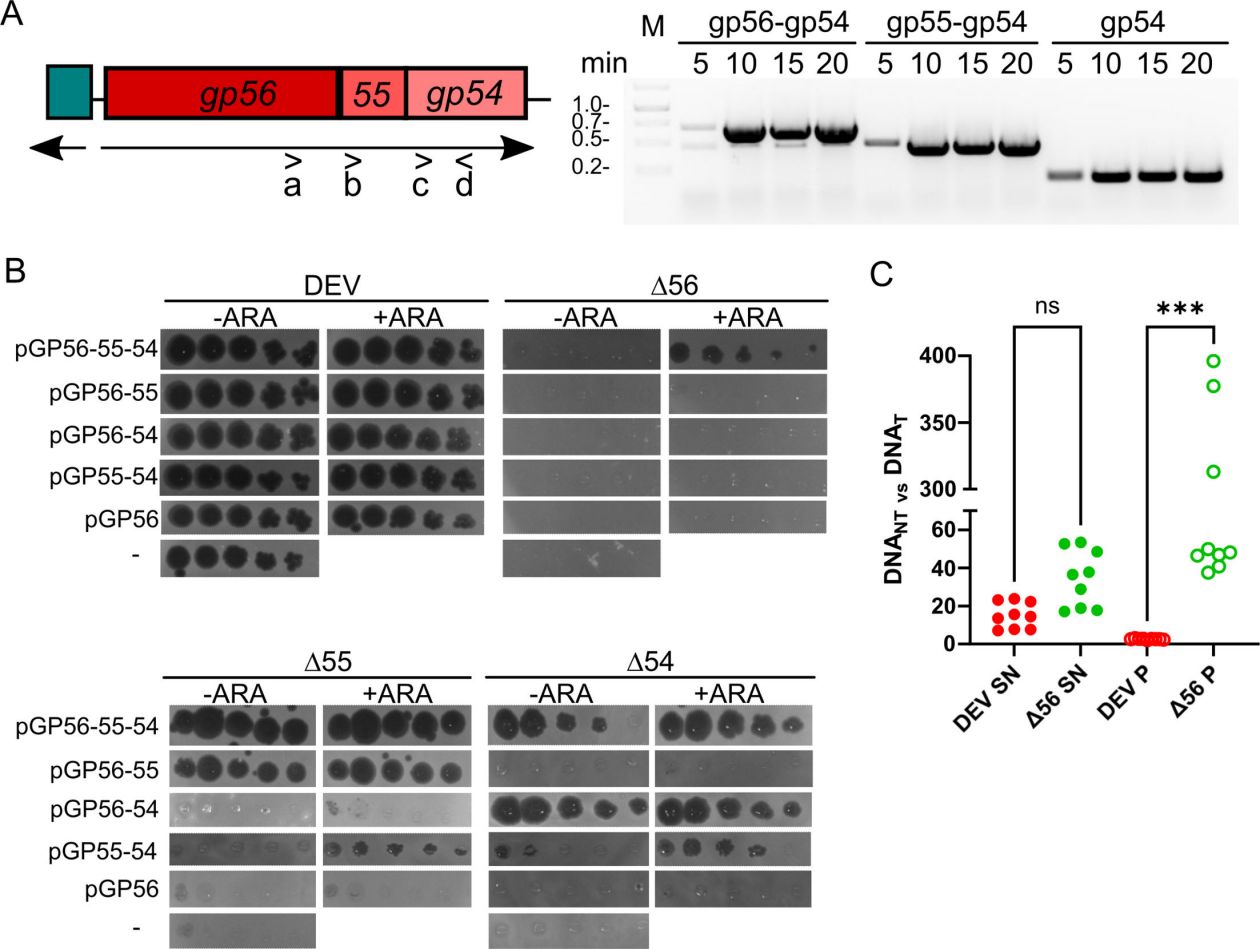
Expression and essential function of *gp56-gp55-gp54* operon. (**A**) RT-PCR analysis of the *gp56-gp55-gp54* operon transcription. The position of the a (4249), b (4250), c (4251), d (4252) oligonucleotides used for PCR amplification is shown on the left of the panel below the arrow indicating the operon transcription direction. gp56-gp54, gp55-gp54, and gp54 amplicons were obtained by RT-PCR with primers a-d, b-d, and c-d, respectively, on RNA extracted at different time points after DEV infection indicated in minutes on top of the lanes. M, MW marker. (**B**) EOP of mutant phages calculated by replica plating of 10-fold serial dilutions (from left to right) of phage lysates. The phages indicated above the panels were replica-plated onto PAO1 carrying the plasmids listed on the left. -, no plasmid. The plates were incubated for 16 h at 37°C. (**C**) DNA release from DEV particles. qPCR analysis of phage DNA content in pellets (P) and supernatants (SN) obtained after PEG- precipitation of phage lysates. DNA_NT_ vs DNA_T_, ratio between DNA amount in samples untreated (NT) vs treated (T) with DNase I. Significance of the difference was estimated with one-way analysis of variance and Šídák’s multiple comparisons test. ns, not significant; ***, *P* = 0.0009.

It has been proposed that several *E. coli* siphoviruses of the *Drexlerviridae* family may utilize LptD as their secondary receptor recognized by a RBP encoded by one of two genes flanking a gene homologous to phage λ *gpJ*, which encodes the distal tail fiber located at the tail tip (21). Interestingly, the final 70% of DEV gp56 short fiber has around 30% identity to gpJ-like proteins of *Drexlerviridae* (Table S1). According to genomic sequence analysis, the *gp56* gene is part of an operon with *gp55* and *gp54*, with the start codons of *gp55* and *gp54* overlapping the stop codons of *gp56* and *gp55*, respectively. Reverse transcription-PCR (RT-PCR) analysis confirmed that the *gp56*, *gp55*, and *gp54* genes are co-transcribed into a single mRNA (Fig. 4A). gp54 was identified as a structural protein according to mass-spectrometry analysis of DEV particles, whereas the identification of gp55 as a structural protein was uncertain (20). Both gp54 and gp55 are highly conserved among *Litunaviruses* (Tables S2 and S3), and gp54 and gp55 orthologs were reported as structural proteins of LIT1, a *Litunavirus* highly similar to DEV (20, 30).

To assess whether the proteins encoded by this operon, and in particular gp56, are essential, we tested the effect of Δ56 deletion on DEV propagation. This deletion removed the whole *gp56* open reading frame (ORF), including a 45 bp long portion of the upstream 5′ untranslated region (UTR) and the first seven codons of *gp55*. The mutant phage did not form plaques on PAO1, whereas it grew in PAO1 carrying a plasmid with the entire *gp56-gp55-gp54* operon. Conversely, plasmids carrying *gp56* only or in combination with either *gp55* or *gp54* did not restore DEV Δ56 growth (Fig. 4B). This finding indicated that the *gp56*, *gp55*, and *gp54* genes are all essential and the Δ56 deletion, which also involves *gp55*, is polar on the downstream *gp54* gene. We did not further explore this aspect, which could be due to translation coupling between gp55 and gp54 and/or premature transcription termination of the poorly translated transcript.

To confirm the conclusion that *gp54* and *gp55* are essential genes, we constructed DEV Δ54 and Δ55 mutants, which carry deletions that eliminate only the *gp54* or the *gp55* ORF, respectively, and tested their growth. We found that the Δ54 and Δ55 mutants grew only if *gp54* or *gp55*, respectively, were expressed from complementing plasmids (Fig. 4B). This confirmed the essentiality of *gp54* and *gp55*.

### The *gp56-55-54* operon controls tail closure

To assess whether the function of the proteins encoded in the *gp56-gp55-gp54* operon, and in particular of gp56, is to control tail plugging, we tested the effect of Δ56 deletion on DNA leakage by phage particles. DEV and DEV Δ56 virions were incubated for 3 h at 37°C and pelleted by PEG-NaCl precipitation. The supernatants and pellets were analyzed by real-time quantitative PCR (qPCR) using phage-specific oligonucleotides before and after DNase I treatment. The enzyme can degrade phage DNA unpackaged or even partially leaked from phage particles, but not the DNA fully packaged inside the phage head. As shown in Fig. 4C, DNase I treatment significantly reduced the DNA content in both supernatants (median DNA decrease of around 14- and 36.5-fold between treated (T) and untreated (NT) samples of DEV and DEV Δ56, respectively). In contrast, the impact of DNase I digestion on DEV DNA in the pellets was much lower (a median 2.6-fold decrease between treated and untreated samples). These results were anticipated, as supernatants are expected to contain unpackaged DNA, whereas pellets are enriched with DNA packaged in phage heads. On the other hand, DEV Δ56 DNA in the pellet was highly susceptible to DNase I digestion (a median 48-fold decrease between T and NT samples), suggesting that the phage genome leaked from the virions.

To strengthen this observation, we assessed the susceptibility of DEV Δ56 virion DNA to digestion with *Bam*HI, which recognizes 16 sites in the DEV genome. DEV Δ56 virions produced in PAO1 (defective virions) or PAO1 expressing *gp56-gp55-gp54* from a plasmid (complete virions) were incubated for 6 h at 37°C with the restriction enzyme, and the phage DNA was analyzed by Southern blotting using a DEV-specific probe (Fig. S1). We found that the DNA from complete virions remained mostly undigested, whereas the DNA from defective virions was completely digested. Overall, these results indicate that gp56 controls the sealing of the phage tail.

### *gp54* mutations suppress DEV defective growth on the *galU lptD*^Δ33^ mutant

**Fig 5:**
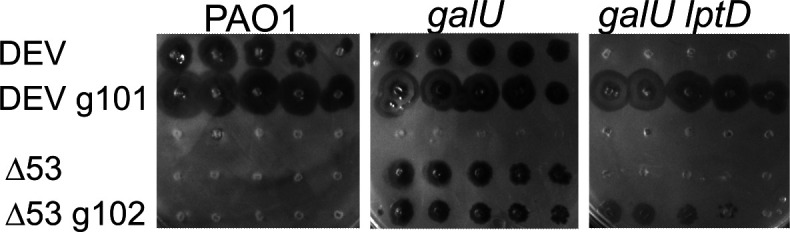
*gp54* mutations suppress DEV defective growth on *galU lptD^Δ33^*. Replica-plating of 10-fold serial dilutions (from left to right) of the phages indicated on the left on PAO1 and PAO1 *galU* and *galU lptD*^Δ33^ (indicated as *galU lptD*) mutants. The plates were incubated for 16 h at 37°C.

We then asked whether, like in *Drexlerviridae*, the *gp54* or *gp55* genes proximal to the *gpJ*-like *gp56* may encode the second RBP of phage DEV. To answer this question, we examined whether phage mutants that regained the ability to grow on the *galU lptD*^Δ33^ strain carried mutations in either of these genes. The rationale was that mutations restoring infectivity are likely to occur in the gene encoding the RBP, which physically interacts with the bacterial receptor. To increase the mutation frequency, we exposed a DEV lysate to UV rays before infecting a liquid culture of the PAO1 permissive host. After 20 min, the infected culture was plated on the *galU lptD*^Δ33^ double mutant. The *gp53-gp54-gp55-gp56* ORFs were amplified from a plaque formed by a mutant phage able to infect this strain (i.e., DEV g101; Fig. 5) and sequenced. Three base substitutions were identified: one resulting in the replacement of aspartate at position 799 with asparagine (D799N) in the gp53 protein and the others resulting in the replacement of arginine at position 37 and threonine at position 181 with glycine and proline, respectively (R37G and T181P mutations), in gp54. No mutations were detected in *gp55* and *gp56*. This result was confirmed by whole genome sequencing of the mutant phage that showed that no other mutations were present in the phage genome.

To strengthen this observation, we looked for mutants of DEV Δ53 able to grow in *galU lptD*^Δ33^. We exposed a DEV Δ53 lysate to UV radiation and infected a liquid culture of the *galU* permissive host. The infected culture was plated onto the *galU lptD*^Δ33^ mutant as described previously. One plaque was isolated and tested by replica-plating on the *galU* and the *galU lptD*^Δ33^ mutants, confirming the ability of this phage (i.e., DEV Δ53 g102) to grow in both strains (Fig. 5). The genome of the mutant phage was sequenced and compared with the DEV genome. Besides the Δ*gp53* deletion, two missense mutations were identified in *gp54*, resulting in amino acid substitutions at positions 85 and 186, namely, valine to isoleucine (V85I) and asparagine to threonine (N186T). These data support the hypothesis that gp54 is the RBP for the DEV secondary receptor.

### Cryo-EM studies reveal DEV RBF complex

**Fig 6:**
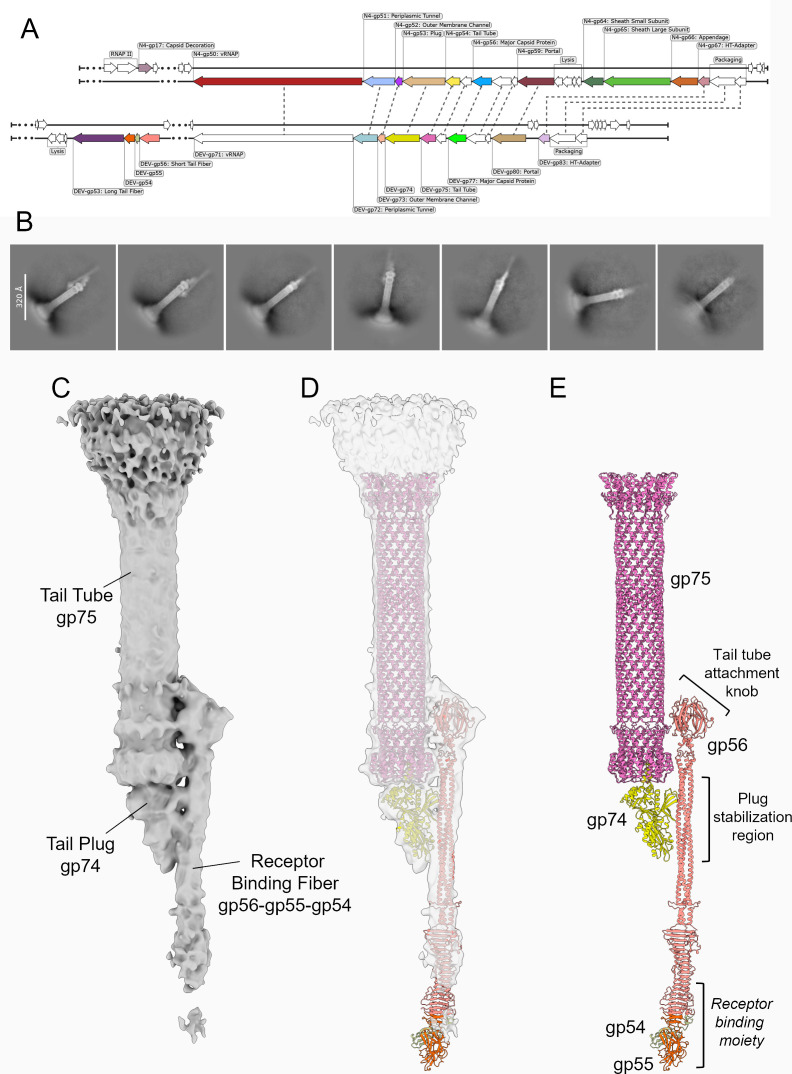
Cryo-EM analysis of DEV tails from DEV Δ53 virions lacking the long tail fiber. (**A**) Comparison of genomic organization between N4 (top) and DEV (bottom) highlights syntenic conservation of the plug gene: *gp53* in N4 and *gp74* in DEV. (**B**) Representative 2D class averages of the DEV tail tip obtained from low-noise micrographs of the Δgp*53* mutant. (**C**) Focused reconstruction of the DEV tail showing the asymmetric interface formed by the tail tube (gp75), tail plug (gp74), and RBF complex (gp56-gp55-gp54). The density, shown in gray, is computed at 8.5 Å resolution (Fig. S2C) and contoured at 4σ. A higher-contour view of this map (14σ) is shown in Fig. S4. (**D**) Semitransparent representation of the density in panel C overlaid with the model of the tail tube gp75 (magenta) and AlphaFold-predicted models of the tail plug gp74 (light blue) and RBF complex (gp56-gp55-gp54). (**E**) Pseudo-atomic model of the DEV tail, comprising gp75, gp74, and the RBF complex (gp56-gp55-gp54), illustrating the overall asymmetry.

Our previous asymmetric reconstruction of the mature DEV virion (20) showed only weak density at the distal tip of the tail tube, insufficient to resolve individual protein components. Based on this observation, we initially hypothesized that the DEV short tail fiber gp56 might function as a tail plug, analogous to the tail needles of other podophages such as P22 (31) and Sf6 (32). However, recent structural studies of phage N4 (33, 34) have identified the gene product 53 (gp53) as the *bona fide* tail plug. This protein, encoded by a gene located downstream of the structural genes for the capsid and tail tube (Fig. 6A), is conserved among N4-like phages, including DEV and LIT1, and has been detected in proteomic analyses of mature virions (30).

To test the hypothesis that gp56 is not the DEV tail plug itself but may interact with the plug to stabilize the packaged genome, we vitrified at high titer DEV Δ53 mutant (20) lacking the long tail fibers (referred to as “appendages” in N4 [35]), and collected an extensive cryogenic electron microscopy (cryo-EM) data set to directly visualize the organization of gp54, gp55, and gp56 in the DEV tail. Applying C12 rotational averaging yielded a 2.9 Å reconstruction of the entire tail, confirming the quality of this data set (Fig. S2A). As expected, asymmetric reconstructions of the tail apparatus from Δ53 virions exhibited substantially reduced noise relative to wt particles (Fig. S2B and S3). A 2D class average of the tail tip (Fig. 6B) revealed an elongated density positioned laterally to the tail tube. Consistently, a low-resolution focused reconstruction of the tail tip showed this elongated lateral density running approximately parallel to the tail tube, accompanied by a roughly triangular density at the distal end of the tail tube (Fig. 6C). These asymmetric density features are visible even at 14σ contour and appear to be equally occupied as the tail tube gp75 (Fig. S4), suggesting they are a genuine structural component of DEV tail. We used AlphaFold3 to predict the structure of the gp56-gp55-gp54 complex, as well as DEV gp74, the putative DEV ortholog of the N4 tail plug gp53. Strikingly, the asymmetric elongated density observed in the focused reconstruction closely resembles an AlphaFold3 (36) model of the DEV gp56 fiber in complex with gp55 and gp54 (Fig. 6D), which we docked into the density and refined using rigid-body fitting. Similarly, the density, occluding the dodecameric tail tube, aligns well with the AlphaFold model of DEV gp74, encoded immediately downstream of the tail tube gene gp75 (Fig. 6A). DEV gp74 exhibits a triangular architecture that caps the tail tube, forming a symmetry-mismatched 12:1 interface (Fig. 6D), as previously observed in phage N4 (33, 34). Visualization of the fitted atomic models suggests that gp74 establishes extensive lateral contacts with the gp56 fiber body (Fig. 6E), explaining the loss of DNA from DEV Δ56 virions (Fig. 4C). In contrast, gp54 and gp55, predicted to bind the C-terminal β-helix of gp56, form the receptor-binding moiety of RBF that remains largely disordered at the resolution of our reconstruction (Fig. 6E). The orientation of gp56 relative to the tail tube is nonetheless unambiguous, despite the limited resolution of the focused reconstruction. Gp56 N-terminal trimeric knob fits precisely into the density immediately above the tail tube tip (Fig. 6E), possibly functioning as an attachment knob. Together, these data provide structural evidence for a previously uncharacterized gp56-gp55-gp54 assembly, which we refer to as the RBF complex.

### AlphaFold3 modeling of RBF-LptD interaction

**Fig 7:**
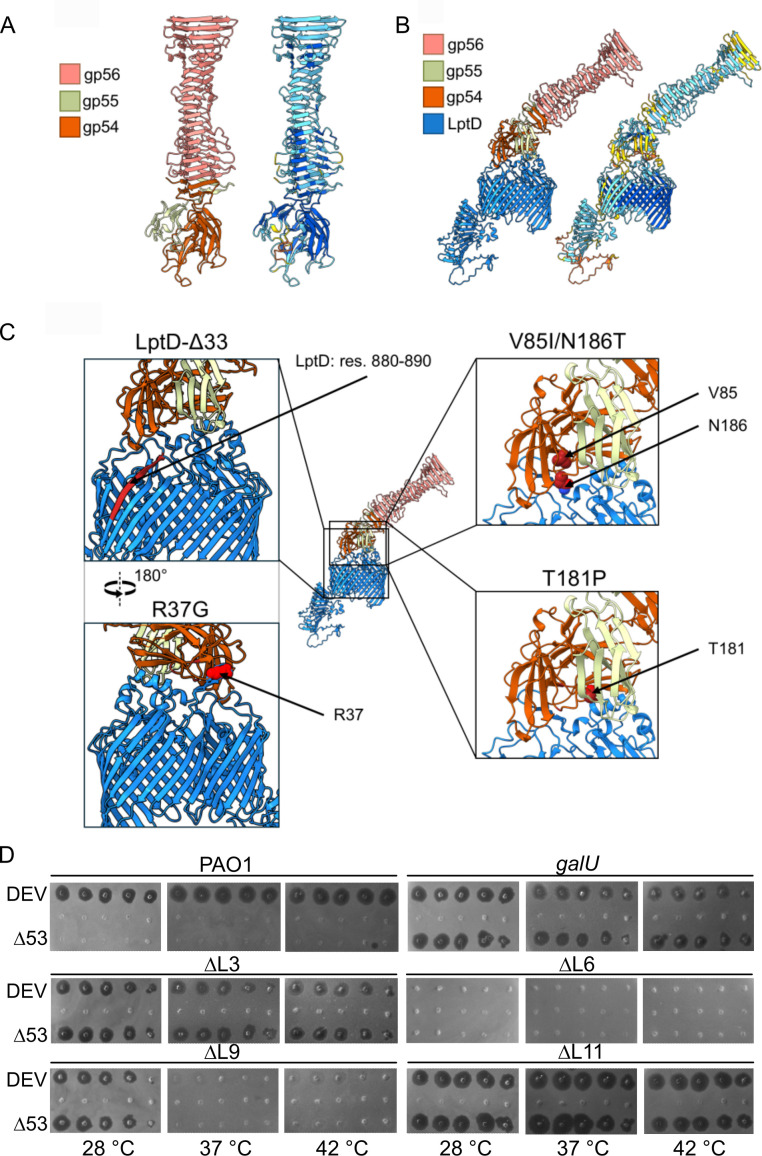
AlphaFold3 predictions of putative DEV receptor binding complex and phenotype of mutants devoid of LptD loops. (**A**) AlphaFold3 prediction of the trimeric gp56 C-terminus (res. 273–429), one copy of full-length gp55 and one copy of full-length gp54. Structure is colored by gene product (left) and according to the AlphaFold3 predicted local distance difference test (pLDDT) (right). The interface predicted template modeling (ipTM) and predicted template modeling (pTM) scores of the complex are 0.8 and 0.81, respectively. (**B**) AlphaFold3 prediction of receptor-binding complex with full-length LptD. Structure is colored by gene product (left) and pLDDT (right). The ipTM and pTM of the structure are 0.35 and 0.48, respectively. (**C**) Residues and regions identified in this study as relevant for adsorption are mapped in red onto the wt receptor and receptor-binding complex prediction. Residues 880–890 missing in LptD-Δ33 (top left); gp54 valine 85 and asparagine 186 (V85 and N186, top right); gp54 arginine 37 (R37, bottom left); gp54 proline 181 (P181, bottom right). (**D**) Replica plating of tenfold serial dilutions (from left to right) of the phages indicated on the left on PAO1 *galU* and *galU lptD* mutants lacking loop 3 (ΔL3), loop 6 (ΔL6), loop 9 (ΔL9), or loop 11 (ΔL11). The plates were incubated for 16 h at the indicated temperatures.

The pseudo-atomic model of the DEV RBF heterotrimer predicts that one gp54 monomer and one gp55 monomer assemble at the tip of a gp56 homotrimer, distal to the capsid, to form a receptor-binding moiety that positions gp54 for recognition of LptD (Fig. 6E). In this structural model, gp56 acts as a structural adaptor between the tail tube and the receptor-binding subunit gp54. In contrast, gp55 stabilizes gp54’s attachment to gp56, possibly contributing to receptor interaction (Fig. 7A). Therefore, AlphaFold3 modeling confirms the experimental observation that gp54 acts as DEV’s RBP, which is exposed to the bacterium’s OM through a structural fiber, gp56, and a potential adaptor, gp55.

We used AlphaFold3 (36) to model the interface between the DEV RBF (gp56-gp55-gp54) complex and its LptD receptor (Fig. 7B). This model positions gp54 directly at the LptD surface, interacting with surface-exposed loops extending from the β-barrel (Fig. S5) (37). Although the predicted interface between the gp54:gp55 complex and LptD has a low confidence score (Fig. 7B) and should therefore be interpreted with caution, the gp54 residues affected by mutations that restore DEV infectivity in the *lptD*^Δ33^ mutant are predicted to lie at this interface, supporting a key role in receptor recognition (Fig. 7C). Based on AlphaFold3 predictions that LptD loop 6 (and possibly loop 3) interacts with gp54, while loops 9 and 11 may interact with gp55 (Fig. S6), we examined the plating efficiency of DEV and DEV Δ53 in *lptD* mutants lacking these loops. As shown in Fig. 7D, loops 3 and 11 were dispensable for phage growth. By contrast, both phages failed to grow in the Δloop6 mutant and showed thermosensitive plating on the Δloop9 mutant, consistent with impaired adsorption in the absence of loop 6 and unstable adsorption to the loop 9 mutant at high temperature.

## DISCUSSION

The adsorption phase of phage infection begins with an initial interaction with a primary receptor, followed by a second step of irreversible binding to a secondary receptor that triggers genome delivery. For some bacteriophages, the receptors are different derivatives or spatially distinct domains of the same molecule, while for others, they are separate molecular entities. The evidence presented in this work places the *P. aeruginosa* DEV phage in the latter group, supporting LptD as the secondary receptor for DEV and gp54 as the RBP recognizing it.

Specifically, we found that the *lptD^Δ33^* mutation renders the deep-rough *galU* mutant resistant to DEV. This deletion removes 11 residues located in loop 13 and β26-strand of LptD, altering the extracellular-facing region of the protein and potentially affecting the lateral gate through which the LPS is transferred from LptD to the OM (37–39) (Fig. S5). *lptD* mutations are known to increase OM permeability by altering its composition, indirectly elevating phospholipid levels, and causing palmitoylation of LPS (40). This raises the possibility that the *lptD^Δ33^* mutation may indirectly inhibit DEV adsorption by altering the OM. Against this hypothesis, DEV resistance is also shown by a mutant lacking the LptD loop 6, which is far from LptD lateral gate and located at the interface with gp54 in the Alphafold3 model. Moreover, the depletion of LpxA, which severely impairs both growth and LPS content, actually enhanced Δ53 phage attachment to PAO1, and ectopic expression of *P. aeruginosa* LptD and LptE in *E. coli* enabled DEV adsorption to *E. coli*, a species distantly related to *P. aeruginosa* and unlikely to produce surface molecules recognized by *P. aeruginosa* phages. However, the adsorption efficiency remained modest, possibly due to inefficient targeting of *P. aeruginosa* LptD to the *E. coli* membrane. Indeed, both wt DEV and the Δ53 mutant show high plating efficiency but low adsorption efficiency on deep-rough PAO1 mutants, suggesting that without the gp53-LPS interaction, phage attachment to the secondary receptor may be unstable and influenced by the experimental procedure used in adsorption assays.

To our knowledge, DEV is the first podovirus and the first *P. aeruginosa* phage proposed to use LptD as a receptor. Genetic evidence from *gp54* mutations suppressing the *galU lptD^Δ33^* resistance to DEV supports a direct interaction between gp54 and LptD, identifying gp54 as the RBP. gp54 is encoded in an operon with the gpJ-like short tail fiber gp56 and gp55. In this study, we provide structural evidence that gp56 functions as a structural module that recruits gp55 and gp54, connecting them to the tail tube while stabilizing the tail plug, which we map to the conserved factor gp74. The asymmetry of the gp56-gp55-gp54 complex, which we designate here as the RBF, highlights the structural complexity of the DEV tail. The RBF represents a unique module that couples receptor recognition to tail unplugging, while permitting evolutionary diversification of the gp54 subunit to accommodate different hosts. The role of gp55, an essential DEV protein, appears to be that of a structural adaptor linking the tail fiber gp56 to the RBP gp54. Moreover, gp55 may help stabilize the interaction with the receptor. This is supported by the thermosensitive growth of DEV in the LptD mutant lacking loop 9, which in the Alphafold3 model is close to gp55.

We propose that DEV and potentially other podoviruses of the *Schitoviridae* family employ an adsorption strategy analogous to that of bacteriophage T5 (and other siphoviruses). These phages sequentially recognize LPS and an OMP, which serve as primary and secondary receptors and are engaged by the lateral tail fibers and the tail tip complex, respectively (21, 41). Specifically, DEV may initiate host recognition through a reversible interaction between its gp53 tail fibers and the O-antigen. This step does not inactivate the phage, consistent with the observation that purified LPS fails to neutralize DEV. Instead, it likely stabilizes virion attachment and positions the phage near its secondary receptor, LptD, which is normally masked by the O-antigen layer in smooth strains (6, 42). Irreversible adsorption then occurs when the RBF complex engages LptD, similar to how T5 binds its final receptor FhuA (41). We propose that this interaction triggers tail-tip unplugging by destabilizing the interaction between the gp56 coiled coil domain and the plug gp74 (Fig. 6E), allowing the release of ejection proteins and the assembly of the DEV ejectosome (20).

Interestingly, although the *Schitoviridae* phages N4 and DEV utilize different host receptors, they appear to share a common strategy in which the primary receptor is transported by the secondary receptor. In fact, the *E. coli* phage N4 relies on the Novel Glycan Receptor (NGR) surface glycan, presumably bound by 12 appendages (gp66), and the NGR transporter NfrA, recognized by the phage gp65 tail sheath, as receptors for infection (43–45). Similarly, N4 employs the sheath protein gp65 to stabilize the tail plug gp53 and engage the NfrA receptor (33, 34), whereas DEV uses the RBF complex to stabilize gp74 and interact with LptD. In both phages, receptor binding via the RBP, gp65 in N4 and gp54 in DEV, triggers destabilization of the plug, leading to release of the ejection proteins (46). This mechanism may enhance adsorption efficiency by concentrating the phage close to the secondary receptor, given that both LPS and NGR are likely densely localized near their respective transporters, LptD and NfrA (47).

The identification of LptD as a phage receptor is potentially relevant for phage therapy, as LptD is an essential and conserved component of the OM, possibly limiting the emergence of phage resistance via receptor modification or loss, or creating an evolutionary trade-off where mutations conferring phage resistance negatively impact cell envelope integrity and, consequently, antibiotic resistance (28). Given the high conservation of gp54 among *Litunaviruses*, we anticipate that other phages in this genus might use LptD as their receptor. However, the O-antigen and outer core effectively shield LptD, preventing phage access. Therefore, the host range of phages that use LptD as a terminal receptor is likely constrained by their ability to recognize and bind the primary receptor, typically the O-antigen. This initial interaction would be the principal determinant of host susceptibility, representing a significant bottleneck in the therapeutic application of LptD-targeting phages. Overcoming the barrier posed by the O-antigen will be essential for enabling these phages to infect a broad spectrum of clinically relevant strains.

## MATERIALS AND METHODS

### Bacteria, bacteriophages, and plasmids

Bacterial strains, bacteriophages, and plasmids are listed in Table S4, oligonucleotides in Table S5. Genome coordinates refer to PAO1 (GenBank accession number NC_002516.2) or DEV (GenBank accession no. MF490238.1). Details about the construction of plasmids, mutant bacterial strains, and phages are provided in Supplemental Methods. DEV deletion mutants were constructed by CRISPR-Cas mutagenesis (20). *lptD* mutants lacking specific extracellular loops were obtained by homologous recombination and sucrose-based selection (48). Bacterial cultures were grown in LB broth at 37°C unless otherwise stated. The cultures were supplemented with 300 µg/mL carbenicillin or 30 µg/mL chloramphenicol for plasmid maintenance. 0.05% or 0.2% (wt/vol) arabinose was added to induce expression from the *araBp* promoter when needed. Depletion of LpxA or LptE in *P. aeruginosa* strains with conditional expression was achieved as described in Supplemental Methods. *E. coli* cultures were maintained with 100 µg/mL ampicillin or 30 µg/mL chloramphenicol, as appropriate.

### Basic techniques of phage handling

Phage lysates were generated as described (16). Methods applied to measure phage EOP, adsorption efficiency, and inactivation by LPS were previously described (19, 26) and are detailed in Supplemental Methods.

### Isolation of DEV-resistant *galU* derivatives

To isolate DEV-resistant *galU* mutants, 0.1 mL of two independent cultures of PAER6b at OD_600_= 1.0 was plated onto two plates spread with 5 × 10^8^ PFU of DEV. Two bacterial colonies were taken from different plates and streaked. Cultures of a sub-clone per each streak were tested as indicators for DEV replica plating to confirm resistance.

### Bacteria and phage genome sequencing

Bacterial genomic DNA was extracted from two DEV-resistant PAER6b derivatives with the Genomic Purification Kit (Gentra System-Puregene). Phage DNA was extracted from 1 mL of high titer filtered lysate (~10¹⁰ PFU/mL) of DEV mutants using a modified phenol:chloroform:isoamyl alcohol method (Supplemental Methods). Bacteria and phage DNA were sequenced on NextSeq 2000 Illumina NGS platform at the SeqCenter (Pittsburgh, PA, USA) applying a sequencing package providing at minimum 400 (for bacteria) or 200 (for phages) Mb of 2 × 151 bp paired reads. Variant calling against PAO1 or DEV reference genomes was performed at the SeqCenter with the breseq software, version 0.36.1 (49). The average depth of sequencing coverage was >300 for bacterial genomes and >3,000 for phage genomes. Besides the new mutation in either *rpoH* or *lptD*, the two bacterial genomes contained already known polymorphisms with respect to the reference genomic sequence (19).

### LPS analysis

LPS was extracted from 1 OD_600_ of exponential bacterial cultures using hot phenol and diethyl ether and analyzed by 18% tricine SDS-PAGE and 12% Bis-Tris SDS-PAGE as previously described (19). The gels were stained using the Pro-Q Emerald 300 staining kit (Thermo Fisher Scientific).

### Phage UV mutagenesis

Fifty microliters of phage suspension at 10^8^ PFU/mL of DEV was irradiated with an UV dose of 10 mJ (λ = 254 nm). Ten microliters of the suspension was mixed with 140 µL of a PAO1 culture at OD_600_ = 0.5 in a 96-well plate. The plate was incubated at 37°C for 120 min and centrifuged 10 min at 4,500 rcf and 4°C. The supernatant was serially diluted and replica-plated onto the susceptible PAO1 and PAER6b hosts and the resistant PAER67 *galU lptD*^Δ33^ strain. Mutant phages forming turbid plaques were isolated on PAER67. DEV Δ53 UV-mutagenesis was similarly performed (Supplemental Methods).

### RT-PCR analysis of *gp56-55-54* operon transcription

RNA extraction from PAO1 cultures was performed at different time points after the infection with DEV and 2 µg of RNA was reverse-transcribed as described (20). 0.5 µL aliquots of the 10 µL cDNA mixtures were PCR-amplified with forward primers 4249, 4250 or 4251, mapping within *gp56*, *gp55*, and *gp54*, respectively, and the reverse primer 4252 mapping within *gp54*. Mock reverse transcription reactions (i.e., without reverse transcriptase) were PCR-amplified to check DNA contamination, which was found to be undetectable.

### DNA leakage assay from DEV Δ56 virions

For the DNase I sensitivity assay, two PAO1 cultures in 10 mL of LB at OD_600_ = 0.1 were infected with DEV or DEV Δ56 with a MOI = 5 and incubated 50 min at 37°C. The infected cultures were pelleted to eliminate uninfected bacteria and cell debris, the supernatants filtered and the phages precipitated overnight at 4°C with 105 g/L of PEG_6000_ and 58 g/L of NaCl. After centrifugation for 30 min at 20,000 × *g* at 4°C, the pellets were resuspended in 1 mL of TN buffer and left 3 weeks at 4°C. Phage suspensions were then incubated 3 h at 37°C with slow shaking. The samples were PEG-precipitated as described before, the pellets resuspended in 1 mL of TN buffer, and the DNA was ethanol-precipitated from 0.5 mL of the supernatants and resuspended in 50 mL of water. Half of each sample was incubated with 10 mg/L DNase I for 30 min at 37°C and for 4 min at 93°C to inactivate the enzyme. The same volume of DNase I-treated (T) and not treated (NT) samples was analyzed by qPCR with primers specific for DEV *gp77* gene (4103–4104). The difference in DNA content was calculated as 2^(CqNT-CqT)^, where CqNT and CqT are the quantification cycles for NT and T samples, respectively. *Bam*HI sensitivity assay is described in Supplemental Methods.

### Cryo-EM analysis of DEV Δ53 virions and AlphaFold modeling

Vitrification and cryo-EM analysis of DEV Δ53 virions is detailed in Supplemental Methods. All AlphaFold models of individual polypeptide chains and complexes were predicted using the AlphaFold3 server (36), as detailed in the Supplemental Methods. Mutation sites discussed in this study were then colored red to roughly localize putative binding interfaces using ChimeraX (50).

## Data Availability

The cryo-EM asymmetric reconstruction of the DEV Δ53 virion tail and the focused reconstruction of the tail tip have been deposited in the Electron Microscopy Data Bank (EMDB) under accession numbers EMD-74075 and EMD-74091. The corresponding atomic models have been deposited in the Protein Data Bank under accession numbers 9ZDW and 9ZE4. All other data supporting the findings of this study are available from the corresponding author upon request.

## SUPPLEMENTAL MATERIAL

Supplemental Material mbio.03561-25-s0001.pdf Supplemental methods, Tables S1 to S5, and Fig. S1 to S6.

ASM does not own the copyrights to Supplemental Material that may be linked to, or accessed through, an article. The authors have granted ASM a non-exclusive, world-wide license to publish the Supplemental Material files. Please contact the corresponding author directly for reuse.
