# Supplementary material for: Pseudomonas aeruginosa DEV phage exploits the essential LptD outer membrane protein as receptor for adsorption
Source: mBio. 2026 Jan 22;17(2):e03561-25. doi: 10.1128/mbio.03561-25 (PMC12892993; doi:10.1128/mbio.03561-25)
Supplement: Supplemental Material — Supplemental methods, Tables S1 to S5, and Fig. S1 to S6. [file mbio.03561-25-s0001.pdf]

SUPPLEMENTARY INFORMATION

Pseudomonas aeruginosa DEV phage exploits the essential LptD outer membrane protein as receptor for adsorption

Jimena Nieto Noblecia[a], Nathan Bellis[b], Cristian A. Antichi[a], Shirin Aminian[a], Francesca Forti[a], Federica A. Falchi[a], Davide Sposato[c], Francesco Imperi[c], Gino Cingolani[b, #], Federica Briani[a, #]

[a]*Università degli Studi di Milano, Dipartimento di Bioscienze, Milan, Italy*

[b]*University of Alabama at Birmingham, Department of Biochemistry and Molecular Genetics, Birmingham, USA*

[c]*Università degli Studi Roma Tre, Dipartimento di Scienze, Rome, Italy*

[#]Corresponding author: gcingola@uab.edu; federica.briani@unimi.it

SUPPLEMENTARY METHODS

Construction of plasmids, mutant bacteria and phages

Plasmids were constructed in *Escherichia coli* DH10b (1) using standard molecular biology techniques. Inserts were verified by PCR and Sanger sequencing prior to introduction into *P. aeruginosa via* electroporation or conjugation. To obtain constructs for generating *lptD* mutants lacking specific LptD extracellular loops, two DNA fragments corresponding to the regions upstream and downstream of each targeted deletion were amplified by PCR and directionally cloned into the *sacB*-based suicide vector pDM4 (2). Primers and restriction enzymes used for PCR and cloning are listed in Tables S4 and S5. All constructs were verified by DNA sequencing. The resulting pDM4 derivatives were transferred into PAO1 *galU* (i.e. PAER6b strain) by conjugation, and transconjugants were selected on plates containing 15 µg/mL nalidixic acid and 375 µg/mL chloramphenicol. Deletion mutants were obtained by homologous recombination and sucrose-based selection as previously described (3), identified by PCR, and finally confirmed by DNA sequencing. DEV deletion mutants were constructed by CRISPR-Cas mutagenesis as described (4). In brief, to obtain DEV Δ56 mutant, a culture of PAO1 carrying plasmid pCas3-13 was grown in LB supplemented with gentamicin (50 µg/ml) and rhamnose (0,1%) to OD_{600} = 0.1. One ml was infected with DEV phage at a multiplicity of infection (moi) = 10 and incubated at 28 °C for 5 min static and 50 min with agitation. The mixture was serially diluted and plated to obtain single plaques on PAO1/pGM2180. After overnight incubation at 28 °C, some plaques were analyzed by replica-plating on PAO1/pGP56-55-54 (permissive strain) and PAO1/pGM931 (non-permissive strain). Four out of 192 spots were formed by phages unable to reproduce without pGP56-55-54. The plaques formed by phages dependent on pGP56-55-54 plasmid were controlled by PCR with oligonucleotides 4043 and 4046 to confirm the presence of the

gp56 deletion. DEV Δ55 was obtained by infecting with DEV a culture of PAO1 carrying plasmid pCas3-21 at moi = 1. One plaque out of 96 tested by replica-plating on PAO1 and PAO1/pGP55-54 as described before carried the Δ55 deletion, as confirmed by PCR with primers 4097-4219 and sequencing of the amplicon. In DEV Δ55, the stop codon of *gp56* overlaps with the start codon of *gp54*, mirroring the arrangement of *gp56-gp55* in DEV genome. DEV Δ54 was obtained by infecting with DEV a culture of PAO1 carrying plasmid pCas3-22 at moi = 0.1. Three plaques out of 240 tested by replica-plating on PAO1 and PAO1/pGP55-54 as described before carried the Δ54 deletion, as confirmed by PCR with primers 4097-4219.

LpxA and LptE Depletion

PAO1 carrying the *lpxA* conditional mutation was cultured overnight at 37 °C in LB medium with (permissive condition) or without (non-permissive condition) 0.2% arabinose. The overnight cultures were diluted in the same media adjusting the OD_{600} to 0.05 and incubated until mid-log phase ($OD_{600} \approx 0.7–0.8$). Cultures were again diluted 1:20 in the same media and incubated further until growth under non-permissive conditions stopped. Equal OD_{600} values were harvested by centrifugation for their use in adsorption assays and for LPS and protein extraction. For LptE depletion, single colonies were incubated in permissive and non-permissive conditions for 24 hours. The cultures were diluted to (theoretical) $OD_{600} = 0.007$ in the same media and incubated overnight at 37 °C. The next day, the cultures were adjusted to $OD_{600} = 0.1$ in the same media and incubated until reaching $OD_{600} \approx 0.7–0.8$, followed by a 1:10 dilution and further incubation until growth in non-permissive conditions slowed down. Equal OD_{600} values were harvested by centrifugation for their use in adsorption assays and for LPS and protein extraction.

Basic techniques of phage handling

Efficiency of plating test. To assess the efficiency of plating (EOP), phage stocks were serially diluted tenfold in 96-well plates, typically beginning with concentrations of 10^8–10^9 plaque-forming units (pfu)/ml in the first well. The dilutions were transferred onto LB agar plates overlaid with 2.5 ml of soft agar combined with 0.2 ml of an overnight culture of *P. aeruginosa* (i.e. the indicator) using either a 48- or 25-pin replicator. The replicators apply approximately 2 µl droplets per spot. *Adsorption assay.* 3 x 10^9 cfu of the bacterial strain under analysis were incubated 10 min at 37°C with 4 x 10^3 pfu of phage in 1 mL of LB. The mixture was centrifuged at 5,000 x g for 10 minutes, and the free phage in the supernatant was titrated using PAO1 or PAER6b permissive strains as indicators. The adsorption efficiency (%) was calculated as [1-(free phage/phage input)] x 100. *DEV inactivation assay.* A total of 10^3 pfu of DEV was incubated with 500 or 50 µg of LPS extracted from PAO1 or PAER6b, or mock-incubated without LPS, for 60 minutes at 37 °C in 0.15 mL of TN buffer (10 mM Tris-HCl, 150 mM NaCl pH 8). Following incubation, the samples were mixed with 0.3 mL of an overnight PAO1 culture and 2.5 mL of soft agar, then poured onto LB agar plates. The EOP was determined by counting plaques after overnight incubation at 37 °C and normalizing the results to those obtained from mock-incubated controls.

DEV Δ53 UV Mutagenesis

We irradiated three 100 µl samples of a 10^9 pfu/ml DEV Δ53 phage suspension with 50 mJ. The samples were pooled and mixed with a 2.7 ml culture at OD_{600} = 0.5 of PAO1 *galU*. The mixture was incubated at 37°C until lysis was visible (~4 hrs). 200 µl of lysate was mixed with 200 µl of PAER67 *galU lptD* in 2.5 ml soft agar, plated on LB, and incubated overnight at 37°C. Single plaques were re-plated on PAER67 to confirm growth.

Genomic DNA extraction from phages

Phage genomic DNA was extracted from 1 ml of high titer filtered lysate (~10^{10} pfu/ml) using a modified phenol:chloroform:isoamyl alcohol method. $MgCl_2$, DNase I and RNase A were added to a final concentration of 12.5 mM, 0.8 U/ml and 0.1 mg/ml, respectively, and the sample was incubated at room temperature for 30 min. Proteinase K, EDTA and SDS at 50 µg/ml, 20 mM and 0.5% (final concentrations) were then added and the lysate was incubated at 55 °C for 1 h with occasional vortexing. The sample was then extracted once with an equal volume of phenol:chloroform:isoamyl alcohol (25:24:1), the aqueous phase was collected after centrifugation at 13,000 rpm for 5 min. The DNA was precipitated with ethanol and high salt. The pellet obtained after centrifugation was resuspended in 50 µL of nuclease-free water.

DNA leakage assay from DEV Δ56 virions

BamHI sensitivity assay. PAO1 and PAO1/pGP56-55-54 cultures were grown in 100 ml of LB up to OD_{600} = 0.04 and infected with DEV Δ56 with a moi= 5. After 60 min at 37 °C, the infected cultures were pelleted to eliminate uninfected bacteria and cell debris, the supernatants filtered and the phages precipitated overnight at 4°C with 105 g/l of PEG_{6000} and 58 g/l of NaCl. After centrifugation for 30 min at 20,000g at 4°C, the pellets were resuspended in 2 ml of MULTI-CORE™ Promega Buffer 1X and incubated 6 hours at 37 °C with *Bam*HI at 100U/ml (final concentration). 0.2 ml were directly sampled for DNA extraction (F samples) and 0.8 ml were PEG-precipitated as described before (P samples) to concentrate virions and DNA molecules longer than 500-700 pb (5). Protein digestion was performed on F and P samples by incubating them with Proteinase K, EDTA and SDS at 50 µg/ml, 20 mM and 0.5% (final concentrations) at 55 °C for 1 h. DNA was prepared by extracting the samples with an equal volume of phenol:chloroform:isoamyl alcohol (25:24:1),

and precipitation with ethanol and high salt. The pellets obtained after centrifugation were resuspended in 20 μL of nuclease-free water and run in 0.8% agarose gels at 80 V for 4.5 h. The gel was transferred onto a Hybond N^+ membrane (Perkin Elmer) by capillary blotting and hybridized with the ^{32}P-radiolabelled oligonucleotide FG4338 (complementary to DEV 16770-16790 region) as described (6). Autoradiographic images of Southern blots were obtained by phosphorimaging with Typhoon FLA 7000 and band densitometry was performed with ImageQuant.

Cryo-EM analysis of DEV Δ53 virions

High titer DEV Δ53 lysate was prepared by infecting PAER6b strain and virions purified as described (4). Virions were vitrified on 200-mesh copper R 2/1 C-Flat holey carbon grids. A total of 3 μL of virions at 1×10^{13} pfu was applied, and grids were blotted using a Vitrobot Mark IV (FEI) for 7 sec at a blot force of 4 before being vitrified immediately in liquid ethane. DEV Δ53 grids were screened in-house at the University of Alabama at Birmingham Cryo-EM Core, on a 200 kV Glacios 2 equipped with a Falcon 4i detector. Micrographs were collected at 120,000x magnification with a pixel size of 1.19 Å, using a total dose of 50 e-/$Å^2$ and a defocus range of -0.5 μm to -2.5 μm. A total of 12,837 movies were collected. EPU software was used for data collection using the fast-positioning mode. All steps of SPA were carried out using cryoSPARC software (7) using Patch Motion Correction and Patch CTF Estimation. Virions were selected using Blob Picker, followed by the removal of junk particles through 2D classification. High-quality picks were used to train a Topaz model that picked total 133,799 DNA-full phage particles. An initial 3D map was generated through ab initio reconstruction with icosahedral(I) symmetry enforced; the Symmetry Expansion job was used to expand I symmetry. A cylindrical mask was created in ChimeraX (8) and resampled to the capsid map to loosely cover an icosahedral vertex. Volume Alignment Tool was used

to move a five-fold vertex into the z-axis and center the particle to that vertex. The re-centered particles were re-extracted whereby another round of 3D classification was used without symmetry enforced to separate out vertices with tails resulting in a single class with phage tails present but unresolved due to heterogeneity. A second round of 3D classification was run on this class, this time with C12 symmetry enforced, resulting in five classes corresponding to the C1 conformation in all five possible conformations with respect to the C5 capsid. Classes were manually inspected and aligned using the Volume Alignment Tools Job by rotating them around the Z-axis by the correct factor of 72 degrees. Duplicates were removed, yielding a total particle number of 73,860 full particles with portals that resolved to a final resolution of 2.91 Å when C12 averaged and to 3.59 Å when the capsid-portal interface was resolved at C1 (Fig. S2A, B). Volume alignment tools were again used to re-center the particles on the poorly resolved tail tip, followed by re-extraction, ideally to crop the dominating signal of the capsid out of the particles. The re-centered and re-extracted particles were 2D-classified, yielding 12,527 particles with a clean tail tube signal, including those with asymmetric elements resolved. These 12,527 particles were then subjected to a 3-class *ab initio* analysis, which yielded one promising class of 8,435 particles. The particles were then refined using non-uniform refinement followed by Local Refinement of re-extracted particles with a larger box size, resulting in a final resolution of 8.58 Å (Fig. S2C).

AlphaFold3 modeling

Previous AlphaFold predictions of gp56 showed high confidence for a trimeric structure comprising a short N-terminal domain, followed by a coiled coil, and ending in a C-terminal β-helix structure (4). This β-helix structure (res. 273-429) was used for all gp56 predictions. Using the AlphaFold3 server (9), various stoichiometric combinations were predicted with three copies of gp56, and the prediction with highest confidence was yielded from 3 copies

of gp56 C-terminus, with 1 copy of gp55 and 1 copy of gp54. This combination was then predicted along with full-length PAO1 LptD. Resulting structures were colored by protein chain identity as well as pLDDT from AlphaFold.

Table S1. Alignment between gp56 and *Drexlerviridae* proteomes

Phage Name	Coverage	Value	Identity (%)	Accession number	Host
vB_PagS_AAS23	59%	9,00E-18	29.52	NC_048137.1	Pantoea
Skenny	63%	2,00E-17	30.74	NC_049841.1	Klebsiella
vB_KppS-Samwise	70%	2,00E-16	29.28	OY639337.1	Klebsiella
vB_KaS-Ahsoka	70%	2,00E-16	29.28	LR881108.1	Klebsiella
vB_KppS-Samwise	70%	2,00E-16	29.28	OY639338.1	Klebsiella
vB_KaS-Gatomon	70%	2,00E-16	29.28	LR881110.1	Klebsiella
vB_KppS-Samwise	70%	2,00E-16	29.28	LR881107.1	Klebsiella
vB_EcoS_PHB17	66%	2,00E-16	29.41	NC_054892.1	Escherichia
IsaakIselin	70%	3,00E-16	25.98	MZ501077.1	Escherichia
JohannLBurckhardt	70%	3,00E-16	29.28	MZ501085.1	Escherichia
Henu7	70%	6,00E-16	29.43	NC_054894.1	Escherichia
vB_KM5a1-KLB26	71%	7,00E-16	27.56	PP556865.1	Klebsiella
mtp21	63%	9,00E-16	28.52	OX335429.1	Klebsiella
mtp20	63%	9,00E-16	28.52	OX335435.1	Klebsiella
mtp18	63%	9,00E-16	28.52	OX335423.1	Klebsiella
mtp17	63%	9,00E-16	28.52	OX335439.1	Klebsiella
vB_KM5a1_KLB19	66%	2,00E-15	27.95	PP526029.1	Klebsiella
vB_KaeD_HazelMika	66%	3,00E-15	28.09	OL539457.1	Klebsiella
vB_EcoS_NBD2	66%	3,00E-15	28.40	KX130668.1	Escherichia
vB_EcoS_NBD2	66%	3,00E-15	28.40	NC_031050.1	Enterobacteria
Sf12	58%	4,00E-14	33.07	NC_047848.1	Shigella
Sd1	58%	1,00E-13	31.91	NC_047847.1	Shigella
KMI8	59%	2,00E-13	27.99	MN101222.1	Klebsiella
Phagiculus	58%	8,00E-13	32.30	OR896821.1	Escherichia
vB_EcoS_IME347	55%	1,00E-12	29.30	NC_047960.1	Enterobacteria
CS01	61%	2,00E-12	25.00	NC_048088.1	Cronobacter
SG01	61%	2,00E-12	25.00	OP120783.1	Cronobacter
BUCT-XGG-1	67%	2,00E-11	27.78	PP069513.1	Escherichia
EC167	76%	1,00E-10	26.71	ON185587.1	Escherichia
vB_Eco_mar001J1	76%	2,00E-10	26.71	NC_048206.1	Escherichia
vB_Eco_mar001J1	76%	2,00E-10	26.71	NC_048204.1	Escherichia
MikeNSara	95%	2,00E-10	22.44	OR896827.1	Escherichia
vB_KpnS_SXFY507	60%	3,00E-10	25.72	ON045001.1	Klebsiella
vB_Kpn_K62PH164C2	48%	4,00E-10	27.44	OY757064.1	Klebsiella
vB_Eco_Sip	95%	4,00E-10	22.44	OU734268.1	Escherichia
NorthRox	95%	4,00E-10	22.44	OR896823.1	Escehrichia
tiwna	95%	4,00E-10	22.44	NC_054896.1	Escherichia
AN_ECEAS	77%	6,00E-10	26.27	PP944860.1	Escherichia
ES	77%	6,00E-10	26.27	OR466728.1	Escherichia

KL3	48%	8,00E-10	26.98	OK019720.1	Klebsiella
vB_Ko_K29PH164C1	53%	9,00E-10	26.94	OY757094.1	Klebsiella
vB_KvaS_F1M1D	54%	1,00E-09	29.72	OL744210.1	Klebsiella
VLCpiD7a	54%	1,00E-09	29.15	ON602741.1	Klebsiella
vB_EcoD_Phunderstruck	74%	2,00E-09	26.52	OL539446.1	Escherichia
vB_KpnS_KpV522	54%	2,00E-09	29.32	NC_047784.1	Klebsiella
BEBK14	67%	2,00E-09	26.90	MN158214.1	Escherichia
WaterSpirit	67%	2,00E-09	26.90	OR896833.1	Escherichia
TSK1	54%	2,00E-09	29.32	NC_048126.1	Klebsiella
PKP126	54%	2,00E-09	29.32	NC_031053.1	Klebsiella
vB_Eco_swan01	74%	3,00E-09	26.52	NC_048202.1	Escherichia
Jahat_MG145	67%	3,00E-09	26.90	MK552105.1	Escherichia
AV105	74%	3,00E-09	26.52	OR352936.1	Escherichia
orkinos	74%	3,00E-09	26.52	MN850586.1	Escherichia
JLBYU19	74%	3,00E-09	26.52	OK272489.1	Escherichia
JLBYU16	74%	3,00E-09	26.52	OK272471.1	Escherichia
JLBYU38	74%	3,00E-09	26.52	OK272481.1	Escherichia
EC125	74%	3,00E-09	26.52	ON185586.1	Escherichia
EC195	74%	3,00E-09	26.52	ON185588.1	Escherichia
tuinn	74%	3,00E-09	26.52	MN850606.1	Escherichia
tunzivis	74%	3,00E-09	26.52	MN850604.1	Escherichia
MatMar	67%	3,00E-09	26.90	OR896829.1	Escherichia
ityhuna	74%	3,00E-09	26.52	MN850582.1	Escherichia
tinuso	74%	3,00E-09	26.52	MN850634.1	Escherichia
vB-Eco-KMB46	74%	3,00E-09	26.52	OR525699.1	Escherichia
tunus	74%	3,00E-09	26.52	OR912983.1	Escherichia
tonn	74%	3,00E-09	26.52	NC_049815.1	Escherichia
tunus	74%	3,00E-09	26.52	NC_049816.1	Escherichia
JLBYU01	74%	3,00E-09	26.52	OK272478.1	Escherichia
MartyMcPhage	74%	3,00E-09	26.52	OR896836.1	Escherichia
ExeQuayColi	74%	3,00E-09	26.52	OR896819.1	Escherichia
vB_EcoS-G3B1	74%	3,00E-09	26.52	MZ234033.1	Escherichia
Tuinin	74%	3,00E-09	26.52	OR896828.1	Escherchia
SECphi27	74%	3,00E-09	26.52	NC_047938.1	Escherichia
JK16	74%	3,00E-09	26.52	NC_049814.1	Shigella
vB_EcoS_XY3	74%	3,00E-09	26.52	MN781674.1	Escherichia
pKP-BS317-1.1	60%	3,00E-09	27.56	OP413832.1	Klebsiella
vB_Kpl_K53PH164C2	54%	4,00E-09	29.32	OY979469.1	Klebsiella
bV_EcoS_AKS96	53%	4,00E-09	29.54	NC_024789.1	Escherichia
vB_EcoS-BECP10	53%	6,00E-09	29.96	MW286156.1	Escherichia
vB_EcoS-UDF157lw	53%	7,00E-09	29.54	OQ243221.1	Escherichia
vB_Kpn_K6PH25C3	54%	8,00E-09	26.78	OY979480.1	Klebsiella
JK06	53%	9,00E-09	29.96	NC_007291.1	Enterobacteria
VLCpiD7b	54%	9,00E-09	28.11	ON602750.1	Klebsiella
bV_EcoS_AHS24	53%	9,00E-09	29.54	NC_024784.1	Escherichia

bV_EcoS_AHP24	53%	9,00E-09	29.54	KF771236.1	Escherichia
JY917	51%	1,00E-08	29.15	NC_049843.1	Klebsiella
e4/1c	53%	1,00E-08	29.54	NC_024210.1	Escherichia
KP13-2	54%	1,00E-08	28.11	OP617742.1	Klebsiella
KP1801	51%	1,00E-08	28.25	NC_049848.1	Klebsiella
bV_EcoS_AHP42	53%	1,00E-08	30.38	NC_024793.1	Escherichia
vB_KvaS_F2M1D	53%	1,00E-08	32.23	OL744213.1	Klebsiella
KP36	54%	1,00E-08	28.03	NC_029099.1	Klebsiella
vB_EcoS_Rogue1	53%	1,00E-08	29.54	NC_019718.1	Enterobacteria
Shelby	54%	1,00E-08	27.20	NC_049846.1	Klebsiella
phiEB49	53%	2,00E-08	29.54	NC_023743.1	Escherichia
phiEB49	53%	2,00E-08	29.54	JF770475.1	Escherichia
phiwza	57%	2,00E-08	27.24	PQ843214.1	Klebsiella
phiJLA23	53%	2,00E-08	29.11	NC_047740.1	Enterobacteria
phiKP26	53%	2,00E-08	29.11	NC_042038.1	Enterobacteriophage
C119	53%	2,00E-08	29.11	NC_042046.1	Escherichia

Table S2. Alignment between gp54 and *Pseudomonas Litunaviruses* proteomes

**Phage Name**	**Coverage**	**E Value**	**Identity (%)**	**Accession number**
vB_PaeP_DEV	100%	1,00E-150	100.00	MF490238.1
vB_PaeP_4032	100%	6,00E-150	99.55	ON815902.1
vB_PaeP_4034	100%	6,00E-150	99.55	ON815903.1
vB_PaeP_4029	100%	6,00E-150	99.55	ON815901.1
vB_PaeP_PYO2	100%	6,00E-150	99.55	MF490236.1
CMS1	100%	7,00E-150	99.55	OM937766.1
vB_Pae_BCPPae_004	100%	8,00E-149	99.10	PV268492.1
PASB7	100%	8,00E-149	98.65	OR509539.1
YH6	100%	2,00E-148	98.21	NC_027388.1
vB_PaeP_MAG4	100%	2,00E-148	98.65	NC_031104.1
vB_PaeP_C2-10_Ab09	100%	6,00E-148	98.65	NC_024140.1
YH30	100%	3,00E-147	98.21	NC_029101.1
PA26	100%	3,00E-147	98.21	NC_041907.1
PA15	100%	3,00E-147	98.21	OM234792.1
Minga_mokiny_4	100%	3,00E-147	98.21	PQ261042.1
vB_Pae1396P-5	100%	3,00E-147	98.21	KX171210.1
vB_Pae575P-3	100%	3,00E-147	98.21	NC_070865.1
PAP02	100%	3,00E-147	98.21	NC_070864.1
phiPA1-3	100%	6,00E-147	98.21	OQ378339.1
RWG	100%	8,00E-147	98.21	KM411958.1
PEV2	100%	8,00E-147	98.21	NC_031063.1
vB_PaP_HN01	100%	9,00E-147	97.76	PP100125.1
VB_PaeS_VL1	100%	1,00E-146	97.31	NC_070862.1
PWJ	100%	1,00E-146	97.31	OR237807.1
vB_Pae_HMKU_23	100%	6,00E-146	96.86	OR988063.1
vB_PaeP_TUMS_P121	100%	1,00E-145	97.31	NC_070863.1
vB_Pae_TUMS_PL	100%	1,00E-145	97.31	PP910670.1
vB_PaeS_TUMS_P81	100%	1,00E-145	97.31	OL519844.1
vB_Pae_TUMS_P11	100%	1,00E-145	97.31	OR424355.1
DL64	100%	3,00E-145	96.86	NC_028885.1
L15	100%	5,00E-141	92.83	OQ992559.1
Ps1.JH	100%	2,00E-133	86.55	PV469326.1
phi176	100%	9,00E-133	86.10	KM411960.1
LP14	100%	1,00E-132	86.10	MH356729.1
LIT1	100%	1,00E-132	86.10	NC_013692.1
Pa2	100%	1,00E-132	86.10	NC_027345.1
vB_PaeP_FMD5	100%	2,00E-132	85.65	PP107937.1
vB_Pae-PA14	100%	2,00E-132	85.65	MZ229661.1
LY218	100%	5,00E-132	85.65	MN906996.1
vB_PaeP_YL1	100%	2,00E-129	84.30	OQ992204.1
BWKH3_R8_1A	100%	2,00E-129	84.30	PQ276738.1
vB_PaeP_YL2	100%	1,00E-128	84.30	OQ992205.1
PaVOA	100%	1,00E-128	84.30	OQ791220.1
PaVOB	100%	1,00E-128	84.30	OQ743452.1
phPLST	100%	3,00E-128	83.41	PV539788.1

vB_PaeP_FBPa1	100%	2,00E-127	83.41	ON857943.1

Table S3. Alignment between gp55 and *Pseudomonas Litunaviruses* proteomes

Phage Name	Coverage	E Value	Identity (%)	Accession number
CMS1	100%	2,00E-62	100.00	OM937766.1
vB_PaeP_DEV	100%	2,00E-62	100.00	MF490238.1
vB_PaeP_MAG4	100%	7,00E-62	98.99	NC_031104.1
vB_PaeP_C2-10_Ab09	100%	1,00E-61	98.99	NC_024140.1
vB_Pae_BCPPae_004	100%	1,00E-61	98.99	PV268492.1
RWG	100%	1,00E-61	98.99	KM411958.1
vB_PaeP_PYO2	100%	1,00E-61	98.99	MF490236.1
PEV2	100%	1,00E-61	98.99	NC_031063.1
vB_PaeP_4032	100%	1,00E-61	98.99	ON815902.1
vB_PaeP_4034	100%	1,00E-61	98.99	ON815903.1
vB_PaeP_4029	100%	1,00E-61	98.99	ON815901.1
YH6	100%	3,00E-61	96.97	NC_027388.1
vB_PaP_HN01	100%	3,00E-61	97.98	PP100125.1
YH30	100%	3,00E-61	97.98	NC_029101.1
PA26	100%	3,00E-61	97.98	NC_041907.1
PA15	100%	3,00E-61	97.98	OM234792.1
Minga_mokiny_4	100%	3,00E-61	97.98	PQ261042.1
vB_Pae_HMKU_23	100%	3,00E-61	97.98	OR988063.1
VB_PaeS_VL1	100%	3,00E-61	97.98	NC_070862.1
PAP02	100%	3,00E-61	97.98	NC_070864.1
PWJ	100%	8,00E-61	96.97	OR237807.1
vB_Pae1396P-5	100%	3,00E-60	96.97	KX171210.1
vB_Pae575P-3	100%	3,00E-60	96.97	NC_070865.1
vB_Pae_TUMS_PL	100%	4,00E-60	96.97	PP910670.1
vB_PaeP_TUMS_P121	100%	2,00E-59	95.96	NC_070863.1
vB_PaeS_TUMS_P81	100%	2,00E-59	95.96	OL519844.1
vB_Pae_TUMS_P11	100%	2,00E-59	95.96	OR424355.1
DL64	100%	8,00E-59	93.94	NC_028885.1
L15	100%	1,00E-56	88.89	OQ992559.1
PASB7	100%	1,00E-55	94.74	OR509539.1
phiPA1-3	100%	2,00E-48	96.83	OQ378339.1
LY218	100%	3,00E-44	75.00	MN906996.1
vB_PaeP_FMD5	100%	1,00E-43	74.00	PP107937.1
vB_PaeP_YL1	100%	1,00E-43	74.00	OQ992204.1
LIT1	100%	1,00E-43	74.00	NC_013692.1
Ps1.JH	100%	1,00E-43	74.00	PV469326.1
Pa2	100%	1,00E-43	74.00	NC_027345.1
phi176	100%	1,00E-43	74.00	KM411960.1
vB_Pae-PA14	100%	1,00E-43	74.00	MZ229661.1
vB_PaeP_YL2	100%	1,00E-43	71.00	OQ992205.1
PaVOA	100%	1,00E-43	71.00	OQ791220.1
PaVOB	100%	1,00E-43	71.00	OQ743452.1

vB_PaeP_FBPa1	100%	2,00E-43	71.00	ON857943.1
BWKH3_R8_1A	100%	2,00E-43	71.00	PQ276738.1
LP14	100%	3,00E-43	74.00	MH356729.1
phPLST	100%	1,00E-42	70.00	PV539788.1

Table S4. Bacterial strains, Bacteriophages and Plasmids

Pseudomonas aeruginosa strains

Strain	Features	Mutation	Reference
PAO1	Reference strain		(10)
PAER6b	PAO1 $galU^{\Delta 1}$	Δ2,215,180[a]	(11)
PAER67	PAER6b $lptD^{\Delta 33}$	Δ(653,860-653,892)[a]	This work
PAER68	PAER6b $rpoH^{164}$	T>G at 421,173[a]	This work
PAO1 *araBp:lpxA*	Δ*lpxA attB*::(*araC-araBp:lpxA*)		(12)
PAO1 *araBp:lptE*	Δ*lptE attB*::(*araC-araBp:lptE*)		(13)
PAER6b ΔL3	PAER6b $lptD^{\Delta loop3}$	Δ(655352-655323)[a]	This work
PAER6b ΔL6	PAER6b $lptD^{\Delta loop6}$	Δ(654972-654920)[a]	This work
PAER6b ΔL9	PAER6b $lptD^{\Delta loop9}$	Δ(654402-654330)[a]	This work
PAER6b ΔL11	PAER6b $lptD^{\Delta loop11}$	Δ(654133-654091)[a]	This work
E. coli JW3606	Δ*waaG::kan*		(14)

Bacteriophages

Name	Features	Mutation position	Reference
DEV	*Litunavirus*		(4, 15)
DEV Δ53	DEV derivative; lacks *gp53*	Δ(29656–32966)[b]	(4)
DEV Δ54	DEV derivative; lacks *gp54*	Δ(32967-33634)[b]	This work
DEV Δ55	DEV derivative; lacks *gp55*	Δ(33635-33934)[b]	This work
DEV Δ56	DEV derivative; lacks *gp56*	Δ(33915-35265)[b]	This work
DEV g101	DEV $gp53^{799}$ $gp54^{37,\ 181}$	C>T at 30,534; T>G at 33,098; T>C at 33,530	This work
Δ53 g102	DEV Δ53 $gp54^{85,\ 186}$	T>G at 33,082; C>T at 33,386	This work
E217	*Pbunavirus*		(15, 16)
E10	Alias NP3*; Pbunavirus*		(15, 17)

Plasmids

Name	Features	Reference
pCas3cRh	It expresses the components of Type I-C CRISPR-Cas system	(18)

pCas3-10	pCas3cRh derivative carrying the annealed 4041-4042 primers cloned in *Bsa*I. It expresses cr-RNA56 targeting *gp56*	This work
pCas3-13	pCas3-10 derivative carrying *gp56* upstream and downstream flanking regions amplified with primers 4043-4105 and 4106-4046, respectively, and cloned in *Bst*Z17I	This work
pCas3-16	pCas3cRh derivative carrying the annealed 4257-4258 primers cloned in *Bsa*I. It expresses cr-RNA54 targeting *gp54*	This work
pCas3-19	pCas3cRh derivative carrying the annealed 4255-4256 primers cloned in *Bsa*I. It expresses cr-RNA55 targeting *gp55*	This work
pCas3-21	pCas3-19 derivative carrying *gp55* upstream and downstream flanking regions amplified with primers 4281-4265 and 4266-4282, respectively, and cloned in *BstZ17I*	This work
pCas3-22	pCas3-16 derivative carrying *gp54* upstream and downstream flanking regions amplified with primers 4277-4309 and 4280-4310, respectively, and cloned in *BstZ17I*	This work
pDM4	Suicide vector for deletion mutagenesis in *P. aeruginosa*; *sacB,* Cm^R	(2)
pDM4Δ*lptD*-L3	pDM4 derivative carrying DNA fragments upstream (amplified with primers L3↑FW- L3↑RV) and downstream (amplified with primers L3↑FW- L11↑RV) of the *lptD* region encoding the extracellular loop 3 (aa 393-402), cloned in the *Apa*I-*Spe*I sites	This work
pDM4Δ*lptD*-L6	pDM4 derivative carrying DNA fragments upstream (amplified with primers L6↑FW- L6↑RV) and downstream (amplified with primers L6↑FW- L11↑RV) of the *lptD* region encoding the extracellular loop 6 (aa 519-536), cloned in the *Apa*I-*Spe*I sites	This work
pDM4Δ*lptD*-L9	pDM4 derivative carrying DNA fragments upstream (amplified with primers L9↑FW- L9↑RV) and downstream (amplified with primers L9↑FW- L11↑RV) of the *lptD* region encoding the extracellular loop 9 (aa 709-733), cloned in the *Apa*I-*Spe*I sites	This work
pDM4Δ*lptD*-L11	pDM4 derivative carrying DNA fragments upstream (amplified with primers L11↑FW- L11↑RV) and downstream (amplified with primers L11↑FW- L11↑RV) of the *lptD* region encoding the extracellular loop 11 (aa 799-813), cloned in the *Apa*I-*Spe*I sites	This work
pGalU	pGM931 derivative carrying the *galU* gene (3299887-3300726)[a] cloned in *Kpn*I under the *araBp* promoter	(11)
pGM931	Cloning vector	(19)
pGM2151	pGM931 derivative. It does not contain the *Pst*I-*Hind*III region of the polylinker	This work
pGM-lptE	*Alias* name, pGM2182. PGS100 derivative carrying the *lptE* gene amplified with primers 4113-4114 and cloned in *Eco* RI-*Pst*I under the *lac* promoter	This work

pGP55-54	*Alias* name, pGM2190. pGM2151 derivative carrying DEV *gp55-gp54* genes amplified with primers 4263-4264 and cloned in the K*pn*I site under the *araBp* promoter	This work
pGP56	*Alias* name, pGM2164. pGM2151 derivative carrying DEV *gp56* gene amplified with primers 4047-4048 and cloned in the K*pn*I site under the *araBp* promoter	This work
pGP56-54	*Alias* name, pGM2191. pGM2151 derivative carrying DEV *gp56-gp54* genes amplified with primers 4047-4265 and 4266-4264 and cloned in the K*pn*I site under the *araBp* promoter. In the construct, the start codon of *gp54* overlaps the stop codon of *gp56*.	This work
pGP56-55	*Alias* name, pGM2189. pGM2151 derivative carrying DEV *gp56-gp55* genes (amplified with primers 4047-4262) between *Kpn*I-*Xba*I under the *araBp* promoter	This work
pGP56-55-54	*Alias* name, pGM2180. pGM2151 derivative carrying DEV *gp56-gp55-gp54* genes amplified with primers 4047-4264 and cloned in the K*pn*I-*Xba*I sites under the *araBp* promoter	This work
PGS100	Cloning vector	(20)
pLptD	*Alias* name, pGM2163. pGM931 derivative carrying the *lptD* gene amplified with primers 4026–4027 and cloned in *Kpn*I under the *araBp* promoter	This work

[a]Coordinates refer to Genbank NC_002516.2
[b]Coordinates refer to Genbank MF490238.1

Table S5. Oligonucleotides

Name	Sequence (from 5’- to 3’-end)
4026	CTCTCTACTGTTTCTCCATGGTACAGGAGATATACATATGGCAGTGAAATCCCTC
4027	GACTCTAGAGGATCCCCGGGTACTTACATAGCTTGATCTTCACGTT
4041	GAAACGGTGTGTACAATGACGGACGCACTGTCGAAGCTGG
4042	GCGACCAGCTTCGACAGTGCGTCCGTCATTGTACACACCG
4043	CGGGGATTCCTTAAGGTAGAGTCCGTGGTCCTCCTATCG
4046	GGTTATGCAGCGGAAAGTACGGTCCAGTTACCGTTAGAAC
4047	CTCTCTACTGTTTCTCCATGGTACCTTTGACCAACTCTGGAGGAAC
4048	GACTCTAGAGGATCCCCGGGTACTCATGCTTGTAGGTTCCCAATG
4097	GTCGGACAACTACCAACCAGGTGTC
4103	AGCCGATGAAGTACAACGAC
4104	AGTCGCACGATCTCTTTGCC
4105	CCCAGCCTATCCACGCAG
4106	CTGCGTGGATAGGCTGGGTTACGACGCCAACGGTAAC
4113	TCAGAATTCATGAAACGTATCCTGACCAGCG
4114	ACTCTGCAGTCACGGGGTGGGGAACTC
4219	GTATTGGGTTCTACCAAGCC
4249	GTGAACAACATCCAGTCGG
4250	CGTCACCGTTGACTCAACC
4251	GCATCGACGAGTTGAATCC
4252	ACCGAACCAAGTGAAGTCC
4255	GAAACGTCAATAACCAGATCCGGTGGTACGACACTTACTG
4256	GCGACAGTAAGTGTCGTACCACCGGATCTGGTTATTGACG
4257	GAAACCCTACCCCTATCAGATCACAGAGCTTGCCAATTTG
4258	GCGACAAATTGGCAAGCTCTGTGATCTGATAGGGGTAGGG
4262	GCCAAGCTGGTCGACTTCATCGGAAGCTCACCACGTTG
4263	CTACTGTTTCTCCATGGTACAGGAGATATACATATGAGTTACGGATTCCGC
4264	GACTCTAGAGGATCCCCGGGTACTTATTCGATGGCCAACAAAGGATGG
4265	TTAATACCGTAAGTCATGCTTGTAGGTTCCCAATGTGC
4266	ATGACTTACGGTATTAAACTGACAAACGAAAACAGTG

4277	CGGGGATTCCTTAAGGTAGGGCACAGGATGGCAGCTTC
4280	GGTTATGCAGCGGAAAGTACCTGAGCCTTGGTCACCTCG
4281	CGGGGATTCCTTAAGGTAGTACAGACCCTGGACGGTAAG
4282	GGTTATGCAGCGGAAAGTAACCGAAGTCCACTCGGAAGTAG
4309	AGTGTACCGTTCATCGGAAGCTCACCACGTTG
4310	TGAGCTTCCGATGAACGGTACACTGCACGCTCC
4338	AGAGCGAACTGATGCCCATCC
L3↑FW	ATAGGGCCCGAGTACGTGATCCACAAG
L3↑RV	CGGGATCCTTGTCGTTAAGGTAGGC
L3↓FW	CGGGATCCCAAGGACCGCTGGCTGTAC
L6↑FW	ATAGGGCCCAAGCGCGGCATGATGC
L6↑RV	CGGGATCCGTCTAGATCTCGATCGAATC
L6↓FW	CGGGATCCTCACTTCAAGGCCTTGCCC
L9↑FW	ATAGGGCCCAATGACGCGCAGCTGG
L9↑RV	CGGGATCCACGGAAGTAGTAAATCTGGC
L9↓FW	CGGGATCCAATGACAGCTGGCGCTCT
L11↑FW	ATAGGGCCCAAGACAGCCTGCCGGT
L11↑RV	CGGGATCCGAGCATCTGCACGATAG
L11↓FW	CGGGATCCACGACATCATCAAGCAGCAC
L11↓RV	GGACTAGTCGAGCTGGCTCTGCATG
ΔL3check	CGCGAAGGTTTCCCGGAC
ΔL6check	TGACGACGGCAGCATCCG
ΔL9check	GCGACTCAACCTAGATCCC
ΔL11check	TCCTCCCGCGGCACATTC

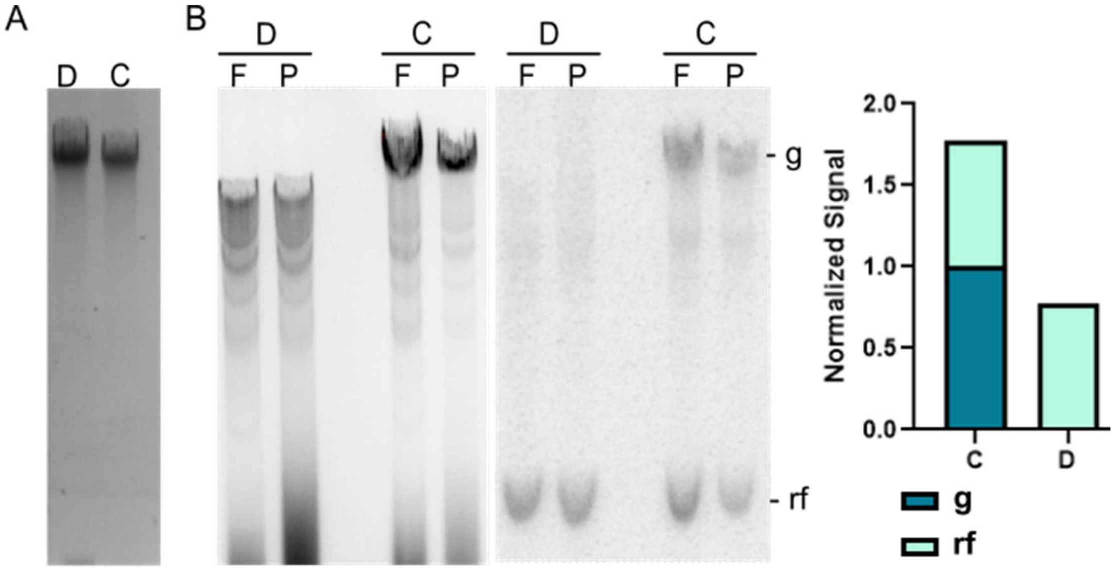

Fig. S1. Effect of gp56 lack on the restriction of DEV DNA. DNA extracted from DEV Δ56 virions prepared upon infection of PAO1 (defective, D) or PAO1 expressing gp56-gp55-gp54 from pGP56-55-54 (complete, C) and incubated with *Bam*HI (B) or not incubated (A). Panels A and B, left. The DNA was run on 0.8% agarose gel and stained with ethidium bromide. B, central panel. The samples were analyzed by Southern blotting with the 4338 radiolabeled oligonucleotide. F and P indicate full and precipitated samples, respectively. g, phage genome; rf, restriction fragment. B, right panel. Bands corresponding to the 1. 64 kb long restriction fragment (rf) and the complete phage genome (g) in the F lanes were quantified with ImageQuant and normalized to the g value in the C sample.

A

C12 Averaged Portal

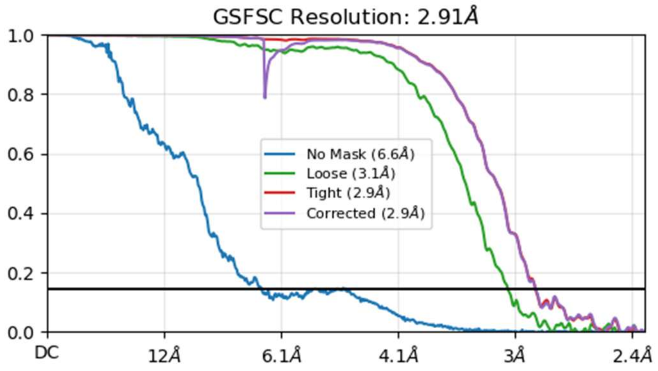

B

Asymmetric Portal-Capsid Interface

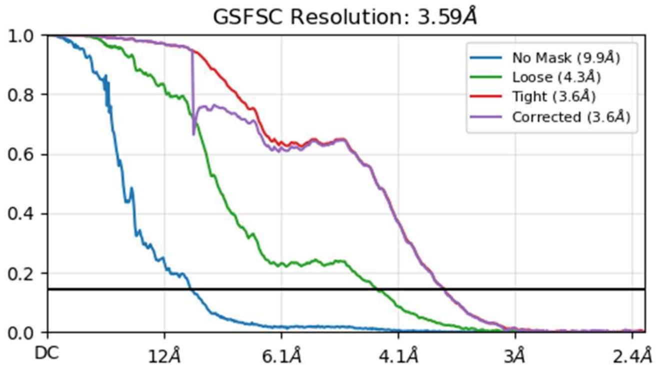

C

Asymmetric gp56-gp75-gp74 Interface

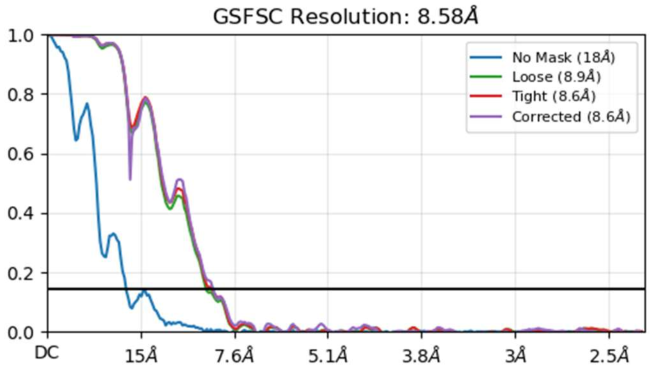

Fig. S2. Fourier shell correlation (FSC) curves for all localized reconstructions generated in this study. Resolutions were determined using the 0.143 FSC cutoff. (A) C12-symmetrized reconstruction of the portal protein, reaching a maximum resolution of 2.91 Å. (B) Asymmetric (C1) reconstruction of the portal–capsid interface (see Fig. S3A), resolved to ~3.59 Å. (C) Low-resolution focused reconstruction of the tail tip (see Fig. S4), showing the asymmetric interface among the DEV tail tube (gp75), tail plug (gp74), and short tail fiber (gp56). The map reaches a maximum resolution of 8.58 Å.

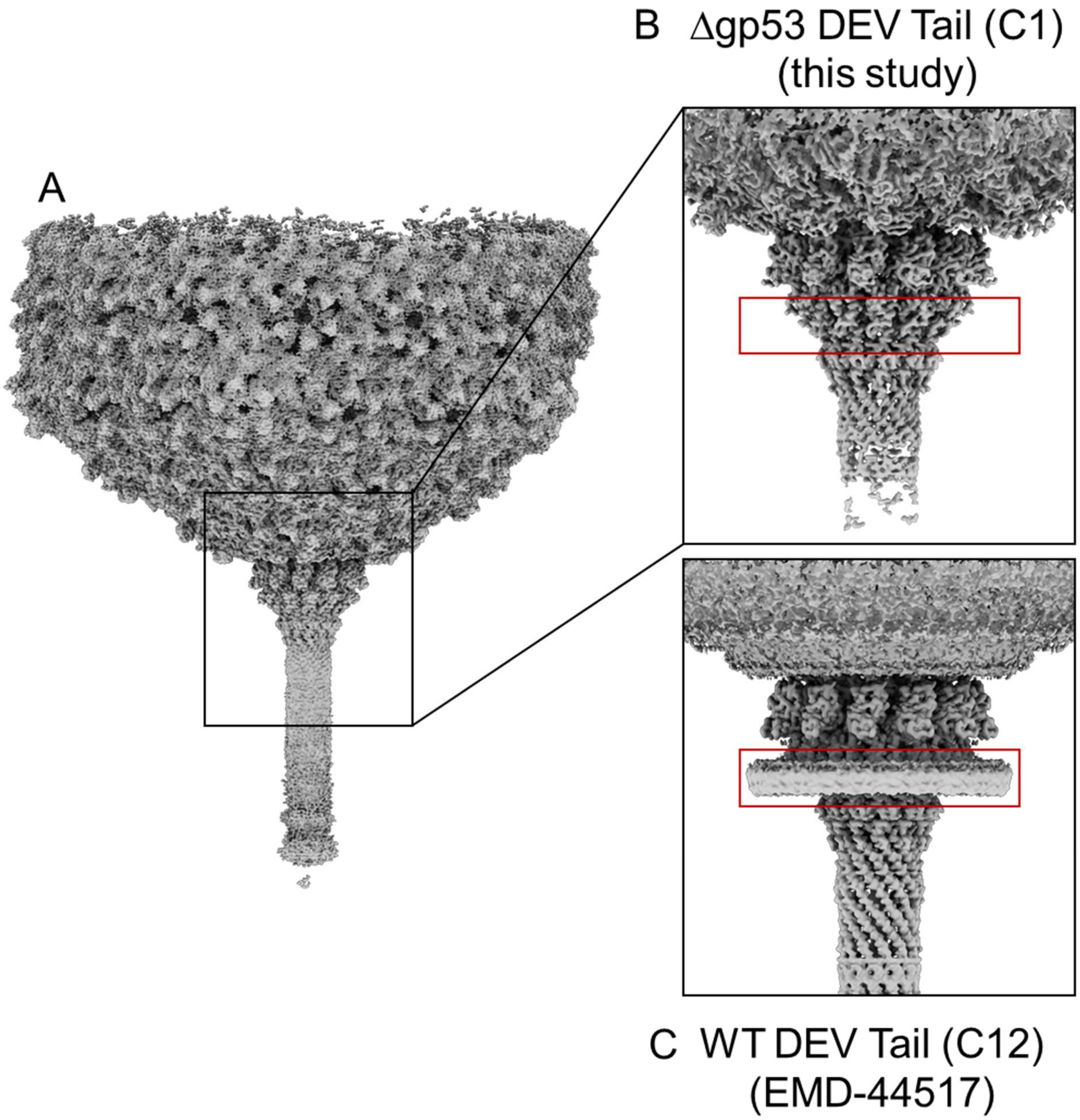

Fig. S3. Reconstruction of DEV Δ53 virions. (A) Section of the asymmetric reconstruction of the DEV Δ53 virion at the portal–capsid interface, resolved to 3.58 Å (the FSC curve for this map is shown in Fig. 2B). (B) Enlarged view of the phage neck region of DEV Δ53. No symmetry was applied. The red box marks the position occupied by the long tail fiber gp53 in the wild-type virion, but absent in this mutant. (C) Corresponding region as in (B) from the C12-symmetrized reconstruction of wild-type (WT) DEV (EMD-44517), showing a ring of smeared density corresponding to the 15 x gp53 fibers (4). All density maps were visualized in ChimeraX (8).

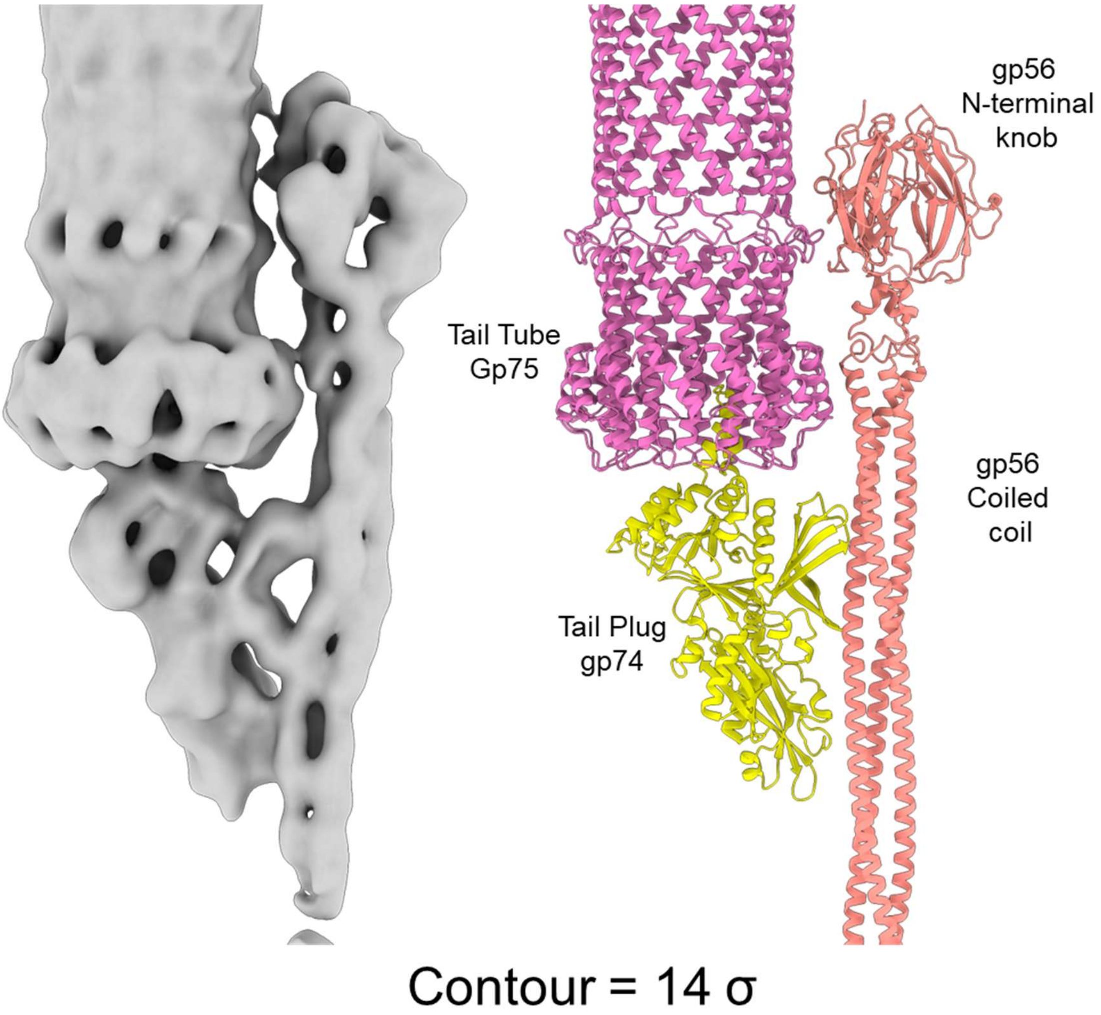

Fig. S4. Focused reconstruction of the DEV gp56–gp75–gp74 interface. Left: the 8.5 Å asymmetric reconstruction of the tail tip contoured at 14σ (FSC curve shown in Fig. S2C). Right: ribbon models of the DEV tail tube gp75 (magenta), tail plug gp74 (yellow), and short tail fiber gp56 (red). The fiber is positioned laterally relative to the tail tube. At high contour levels, only the trimeric knot and coiled-coil region of gp56 are visible. The flexible C-terminal β-helix of gp56, which interacts with gp54 and gp55, is not resolved.

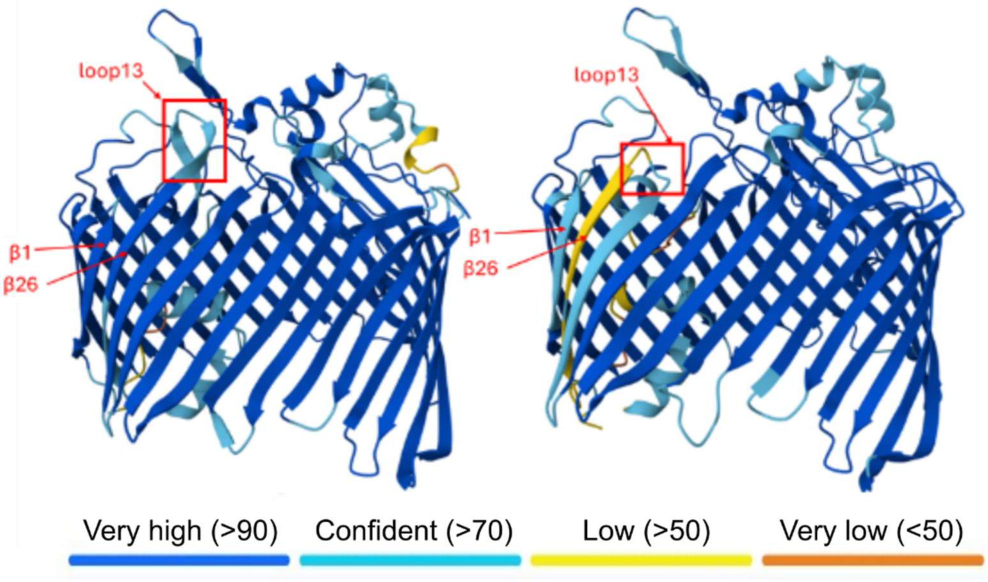

Fig. S5. Alphafold3 prediction of the effect of the l*ptD*^{Δ33} mutation on LptD. The structure of the beta-barrel portion of the *P. aeruginosa* LptD transporter (amino acid 319-924) was predicted with AlphaFold 3 (9). On the left, the wt LptD; on the right, the predicted effect of *lptD*^{Δ33} mutation on LptD structure. β1-β26 regions forming the gate are indicated and loop13 is boxed. Colors indicate the local confidence of the predicted structure calculated as pIDDT. The pTM of both structures is 0.91.

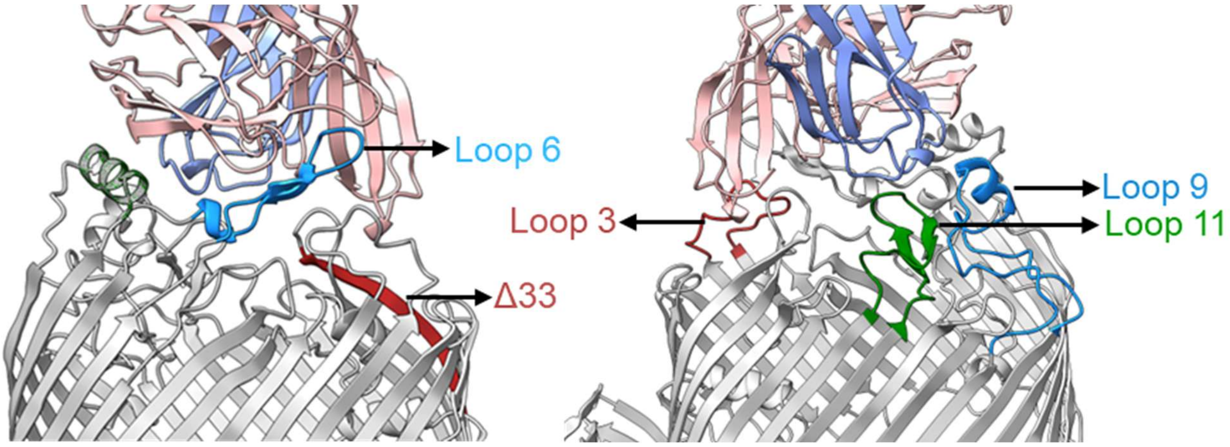

Fig. S6. LptD loops at the interface with DEV RBF. AlphaFold3 prediction of receptor binding complex with full-length LptD (Figs. 7B-C). gp54 is represented in pink, gp55 in light blue, LptD in grey. The region deleted in the *galU lptD*$^{\Delta 33}$ mutant (Δ33) and the loops deleted in this work are indicated.

Supplementary References

1. Grant SGNN, Jessee J, Bloom FR, Hanahan D. 1990. Differential plasmid rescue from transgenic mouse DNAs into *Escherichia coli* methylation-restriction mutants. Proc Natl Acad Sci U S A 87:4645–4649.

2. Milton DL, O'Toole R, Hörstedt P, Wolf-Watz H. 1996. Flagellin A is essential for the virulence of *Vibrio anguillarum*. J Bacteriol 178:1310–1319.

3. Lo Sciuto A, Spinnato MC, Pasqua M, Imperi F. 2022. Generation of Stable and Unmarked Conditional Mutants in *Pseudomonas aeruginosa*. Methods Mol Biol 2548:21–35.

4. Lokareddy RK, Hou C-FD, Forti F, Iglesias SM, Li F, Pavlenok M, Horner DS, Niederweis M, Briani F, Cingolani G. 2024. Integrative structural analysis of *Pseudomonas* phage DEV reveals a genome ejection motor. Nat Commun 15:8482.

5. Lis JT, Schleif R. 1975. Size fractionation of double-stranded DNA by precipitation with polyethylene glycol. Nucleic Acids Res 2:383–390.

6. Sambrook J, Russell DW. 2001. Molecular Cloning: A Laboratory Manual, Third EditionMolecular Cloning: a laboratory a manual,.

7. Punjani A, Rubinstein JL, Fleet DJ, Brubaker MA. 2017. cryoSPARC: algorithms for rapid unsupervised cryo-EM structure determination. Nat Methods2017/02/07. 14:290–296.

8. Meng EC, Goddard TD, Pettersen EF, Couch GS, Pearson ZJ, Morris JH, Ferrin TE. 2023. UCSF ChimeraX: Tools for structure building and analysis. Protein Science 32:e4792.

9. Abramson J, Adler J, Dunger J, Evans R, Green T, Pritzel A, Ronneberger O, Willmore L, Ballard AJ, Bambrick J, Bodenstein SW, Evans DA, Hung CC, O'Neill M, Reiman D, Tunyasuvunakool K, Wu Z, Žemgulytė A, Arvaniti E, Beattie C, Bertolli O, Bridgland A, Cherepanov A, Congreve M, Cowen-Rivers AI, Cowie A, Figurnov M, Fuchs FB, Gladman H, Jain R, Khan YA, Low CMR, Perlin K, Potapenko A, Savy P, Singh S, Stecula A, Thillaisundaram A, Tong C, Yakneen S, Zhong ED, Zielinski M, Žídek A, Bapst V, Kohli P, Jaderberg M, Hassabis D, Jumper JM. 2024. Accurate structure prediction of biomolecular interactions with AlphaFold 3. Nature 630:493–500.

10. Stover CK, Pham XQ, Erwin AL, Mizoguchi SD, Warrener P, Hickey MJ, Brinkman FSL, Hufnagle WO, Kowallk DJ, Lagrou M, Garber RL, Goltry L, Tolentino E, Westbrock-Wadman S, Yuan Y, Brody LL, Coulter SN, Folger KR, Kas A, Larbig K, Lim R, Smith K, Spencer D, Wong GKS, Wu Z, Paulsen IT, Relzer J, Saler MH, Hancock REW, Lory S, Olson M V. 2000. Complete genome sequence of *Pseudomonas aeruginosa* PAO1, an opportunistic pathogen. Nature 406:959–964.

11. Forti F, Bertoli C, Cafora M, Gilardi S, Pistocchi A, Briani F. 2023. Identification and impact on *Pseudomonas aeruginosa* virulence of mutations conferring resistance to a phage cocktail for phage therapy. Microbiol Spectr e0147723.

12. Cervoni M, Ferriero AM, Lo Sciuto A, Guidi F, Babić Jordamović N, Piazza S, Jousson O, Esposito A, Imperi F. 2025. The Genetic Background and Culture Medium Only Marginally Affect the *In Vitro* Evolution of *Pseudomonas aeruginosa* Toward Colistin Resistance. Antibiotics 14:601.

13. Lo Sciuto A, Martorana AM, Fernández-Piñar R, Mancone C, Polissi A, Imperi F. 2018. *Pseudomonas aeruginosa* LptE is crucial for LptD assembly, cell envelope integrity, antibiotic resistance and virulence. Virulence 9:1718–1733.

14. Ebbensgaard A, Mordhorst H, Aarestrup FM, Hansen EB. 2018. The Role of Outer Membrane Proteins and Lipopolysaccharides for the Sensitivity of *Escherichia coli* to Antimicrobial Peptides. Front Microbiol 9:2153.

15. Forti F, Roach DR, Cafora M, Pasini ME, Horner DS, Fiscarelli E V., Rossitto M, Cariani L, Briani F, Debarbieux L, Ghisotti D. 2018. Design of a broad-range bacteriophage cocktail that reduces *Pseudomonas aeruginosa* biofilms and treats acute infections in two animal models. Antimicrob Agents Chemother 62:e02573-17.

16. Li F, Hou C-FD, Lokareddy RK, Yang R, Forti F, Briani F, Cingolani G. 2023. High-resolution cryo-EM structure of the *Pseudomonas* bacteriophage E217. Nat Commun 14:4052.

17. Chaudhry WN, Concepcion-Acevedo J, Park T, Andleeb S, Bull JJ, Levin BR. 2017. Synergy and Order Effects of Antibiotics and Phages in Killing *Pseudomonas aeruginosa* Biofilms. PLoS One 12:e0168615.

18. Csörgő B, León LM, Chau-Ly IJ, Vasquez-Rifo A, Berry JD, Mahendra C, Crawford ED, Lewis JD, Bondy-Denomy J. 2020. A compact Cascade-Cas3 system for targeted genome engineering. Nat Methods 17:1183–1190.

19. Delvillani F, Sciandrone B, Peano C, Petiti L, Berens C, Georgi C, Ferrara S, Bertoni G, Pasini MEME, Dehò G, Briani F. 2014. Tet-Trap, a genetic approach to the identification of bacterial RNA thermometers: application to *Pseudomonas aeruginosa.* RNA 20:1963–1976.

20. Sperandeo P, Pozzi C, Dehò G, Polissi A. 2006. Non-essential KDO biosynthesis and new essential cell envelope biogenesis genes in the *Escherichia coli yrbG-yhbG* locus. Res Microbiol 157:547–558.
